# Light-harvesting chlorophyll *a*/*b*-binding proteins, positively involved in abscisic acid signalling, require a transcription repressor, WRKY40, to balance their function

**DOI:** 10.1093/jxb/ert307

**Published:** 2013-09-27

**Authors:** Rui Liu, Yan-Hong Xu, Shang-Chuan Jiang, Kai Lu, Yan-Fen Lu, Xiu-Jing Feng, Zhen Wu, Shan Liang, Yong-Tao Yu, Xiao-Fang Wang, Da-Peng Zhang

**Affiliations:** ^1^Bioinformatics and Systems Biology Laboratory of the Ministry of Education, Scholl of Life Sciences, Tsinghua University, Beijing 100084, PR China; ^2^Institute of Medicinal Plant Development, Chinese Academy of Medical Sciences & Peking Union Medical College, Beijing 100193, PR China

**Keywords:** Abscisic acid signalling, *Arabidopsis thaliana*, light-harvesting chlorophyll *a/b*-binding protein, post-germination growth, seed germination, WRKY40 transcription factor.

## Abstract

The light-harvesting chlorophyll *a*/*b*-binding (LHCB) proteins are the apoproteins of the light-harvesting complex of photosystem II. In the present study, we observed that downregulation of any of the six *LHCB* genes resulted in abscisic acid (ABA)-insensitive phenotypes in seed germination and post-germination growth, demonstrating that LHCB proteins are positively involved in these developmental processes in response to ABA. ABA was required for full expression of different LHCB members and physiologically high levels of ABA enhanced *LHCB* expression. The LHCB members were shown to be targets of an ABA-responsive WRKY-domain transcription factor, WRKY40, which represses *LHCB* expression to balance the positive function of the LHCBs in ABA signalling. These findings revealed that ABA is an inducer that fine-tunes *LHCB* expression at least partly through repressing the WRKY40 transcription repressor in stressful conditions in co-operation with light, which allows plants to adapt to environmental challenges.

## Introduction

The light-harvesting chlorophyll *a*/*b*-binding (LHCB) proteins are the apoproteins of the light-harvesting complex of photosystem II (PSII). LHCB proteins are normally associated with chlorophyll and xanthophylls and serves as the antenna complex. These antenna complexes absorb sunlight and transfer the excitation energy to the core complexes of PSII in order to drive photosynthetic electron transport (Jansson, 1994, 1999). The PSII outer antenna LHCB proteins are important components of the major light-harvesting complex, and consist of minor antenna complexes LHCB4 (CP29), LHCB5 (CP26), and LHCB6 (CP24) and major antenna complexes that comprise homo- and heterotrimers of LHCB1, LHCB2, and LHCB3 (Jansson, 1994, 1999).

These chloroplast/thylakoid proteins are encoded by nuclear genes. Expression of the *LHCB* genes is tightly regulated by developmental cues as well as by multiple environmental signals. Several developmental signals have been reported to be involved in the regulation of *LHCB* expression, includingthe chloroplast retrograde signal (review by Nott *et al.*, 2006) and circadian clock (Paulsen and Bogorad, 1988; Strayer *et al.*, 2000; Alabadi *et al.*, 2001; Thain *et al.*, 2002; Andronis *et al.*, 2008; Pruneda-Paz *et al.*, 2009; De Montaigu *et al.*, 2010; Pruneda-Paz and Kay, 2010; Thines and Harmon, 2010). It is well established that light is the most important environmental signal to regulate *LHCB* expression (Silverthorne and Tobin, 1984; Sun and Tobin, 1990; Millar and Kay, 1996; Peer *et al.*, 1996; Weatherwax *et al.*, 1996; Yang *et al.*, 1998; Humbeck and Krupinska, 2003; Staneloni *et al.*, 2008; De Montaigu *et al.*, 2010; Pruneda-Paz and Kay, 2010; Thines and Harmon, 2010). Several environmental stresses such as oxidative stress have been reported to affect *LHCB* expression (Nott *et al.*, 2006; Staneloni *et al.*, 2008).

The phytohormone abscisic acid (ABA), which is an important plant signal in response to various environmental stress conditions, has been reported to play a negative role in the regulation of *LHCB* expression (Bartholomew *et al.*, 1991; Chang and Walling, 1991; Weatherwax *et al.*, 1996; Staneloni *et al.*, 2008). Exogenously applied ABA downregulates *LHCB* gene expression in tomato leaves (Bartholomew *et al.*, 1991), *Arabidopsis* seedlings (Staneloni *et al.*, 2008), *Lemna gibba* cells grown on liquid medium (Weatherwax *et al.*, 1996), and developing seeds of soybean (Chang and Walling, 1991). Downregulation of *LHCB* expression by high light is likely to be mediated by changes in ABA concentrations (Weatherwax *et al.*, 1996). However, a recent report showed that the treatment of the 6-d-old *Arabidopsis* seedlings with low levels of ABA (from 0.125 to 1 µM) enhanced *LHCB1.2* mRNA levels (Voigt *et al.*, 2010). Additionally and importantly, previous studies showed that members of the LHCB family play an important role in plant adaptation to environmental stresses (Andersson *et al*., 2001, 2003; Ganeteg *et al.*, 2004; Kovacs *et al.*, 2006; Xu *et al.*, 2012). Thus, it isnecessary to determine whether ABA plays a positive or negative role in *LHCB* expression and how ABA functions in this cell signalling process, which is of importance for understanding the mechanisms of functions of LHCB proteins involved in plant stress signalling.

Recently, we showed that LHCB members are positively involved in ABA signalling in stomatal movement and the plant response to drought (Xu *et al.*, 2012). In the present study, we showed that LHCB members positively regulate seed germination and post-germination growth in response to ABA. We observed that ABA was required for full expression of different LHCB members and that physiologically high levels of ABA enhanced*LHCB* expression, and furthermore, we have provided evidence to show that ABA functions through an ABA-responsive WRKY transcription factor, WRKY40, which represses *LHCB* expression to balance the function of the LHCB members in ABA signalling.

## Materials and methods

### Plant materials and growth conditions

*Arabidopsis thaliana* ecotype Columbia (Col-0) was used in the experiments. The *wrky40-1* (stock number: ET5883, with Ler ecotype as background) was obtained from Cold Spring Harbor Laboratory gene and enhancer trap lines, which contain a Ds transposon inserted within the second exon of *WRKY40* (*Arabidopsis* genomic locus tag: At1g80840). The *wrky40-1* mutation was transferred from its background Ler ecotype into the Col-0 ecotype by backcrossing, as described previously (Shang *et al.*, 2010). The *wrky18-1* mutant (SALK_093916) is a T-DNA insertion knockout mutant with a T-DNA insertion within the first exon in *WRKY18* (At4g31800), which was isolated from the Col-0 ecotype. Both mutants were previously identified as null alleles in their respective genes (Shang *et al.*, 2010)and were obtained from the *Arabidopsis* Biological Resource Center (ABRC). The seeds of the ABA-deficient mutant *aba2* (CS156: *aba2*-*1*, with the Col-0 ecotype as background) and other mutants *abi5* (CS8105: *abi5-1*), *lhcb1.1* (SALK-134810), *lhcb2.2* (SALK-005614), *lhcb3* (SALK-036200), *lhcb4.4* (SALK-032779), *lhcb5* (SALK-139667), and *lhcb6* (SALK-074622) were also obtained from ABRC. The *wrky40 wrky18*, *lhcbs* and *wrky40 lhcb* double mutants was generated by genetic crosses and identified by PCR genotyping as previously described (Shang *et al.*, 2010).

Plants were grown in a growth chamber at 19–20 °C on Murashige–Skoog (MS) medium (Sigma, St Louis, MO, USA) at ~80 µmol photons m^–2^ s^–1^, or in compost soil at about 120 µmol photons m^–2^ s^–1^over a 16h photoperiod.

### Effects of ABA treatment on *LHCB* mRNA and protein levels

Three-day-old young seedlings were transferred to MS medium supplemented with ABA at the indicated concentrations and continued to grow for 2 weeks before sampling. Two-week-old seedlings were also transferred to soil to continue to grow for 3 weeks, and these 5-week-old plants were sprayed with ABA solutions at the indicated concentrations and sampled 5h later for analysis.

### Real-time PCR analysis

Total RNA was isolated using a Total RNA Rapid Extraction kit (BioTeke), treated with RNase-free DNase I (Takara) at 37 °C for 30min to degrade genomic DNA and purified using an RNA Purification kit (BioTeke). A 2 µg aliquot of RNA was subjected to first-strand cDNA synthesis using Moloney murine leukemia virus reverse transcriptase (Promega), and an oligo(dT)_21_ primer. The primers used for real-time PCR are listed in Supplementary Table S1 at *JXB* online. Analysis was performed using a BioRad Real-Time System CFX96TM C1000 Thermal Cycler (Singapore).

### Protein extraction and immunoblotting

Extraction of the *Arabidopsis* total proteins was performed essentially according to procedures proposed by the LHCBantibody supplier Agrisera (Stockholm, Sweden). The plant tissues were frozen in liquid N_2_, ground in a pre-chilled mortar with a pestle to a fine powder and transferred to a 1.5ml tube. The extraction buffer consisted of 50mM Tris/HCl (pH 7.5), 150mM NaCl, 1mM EDTA, 0.1% (v/v) Triton X-100, 10 % (v/v) glycerol, and 5 µg ml^–1^ protein inhibitor cocktail. The extraction buffer was added to the tube (buffer:sample ratio of 4:1), which was immediately frozen in liquid N_2_. The mixture was carefully subjected to sonication until the sample was just thawed, and was refrozen immediately in liquid N_2_ to avoid heating. The sonication step was repeated three times. The mixture was centrifuged for 3min at 10 000*g* to remove insoluble material and unbroken cells, and the supernatant was transferred to a new tube for use. SDS-PAGE and immunoblotting assays were done essentially according to our previously described procedures (Wu *et al.*, 2009; Shang *et al.*, 2010). Specific antibodies against LHCB1, LHCB2, LHCB3, LHCB4, LHCB5, and LHCB6 were purchased from Agrisera.

### WRKY40/LHCB promoter interaction tested with yeast one-hybrid assays

Yeast one-hybrid assays were performed as described previously (Shang *et al.*, 2010) with a Matchmaker™ One-Hybrid Library Construction & Screening kit (Clontech) using the AH109 yeast strain. The primers used for cloning the *LHCB* promoters are listed in Supplementary Table S1. The promoter DNA fragment was subcloned into the *Sma*I/*Mlu*I sites of the pHIS2 vector. The one-hybrid assays were performed using the AH109 yeast strain according to the manufacturer’s instructions. Yeast cells were co-transformed with pHIS2 bait vector harbouringthe promoter of targetgenes and pGADT7 prey vector harbouringthe open reading frame of *WRKY40*, as described previously (Shang *et al.*, 2010). As negative controls, the yeast cells were co-transformed with the combination of pGADT7-*WRKY40* and empty pHIS2 vector, empty pGADT7 vector and pHIS2 harbouring the corresponding promoter, or two empty vectors pGADT7 and pHIS2. Transformed yeast cells were first grown in SD–Trp–Leu medium to ensure that the yeast cells were successfully co-transformed, and the co-transformed yeast cells were then grown on SD–Trp–Leu–His medium plates. The SD–Trp–Leu or SD–Trp–Leu–His medium was supplemented with 3-amino-1,2,4-triazole (Sigma) at 25mM (for WRKY40–*LHCB1*, WRKY40–*LHCB2*,orWRKY40–*LHCB5*promoter interaction) or 10mM (for WRKY40–*LHCB3* or WRKY40–*LHCB6*promoter interactions). The plates were then incubated for 3 d at 30 °C.

### Chromatin immunoprecipitation (ChIP) assays

ChIP assayswere performed essentially as described previously (Saleh *et al.*, 2008; Shang *et al.*, 2010). Two-week-old seedlings were sampled for the assays. The WRKY40-specific antibody against WRKY40N (an N-terminal truncated form of WRKY40), produced as described previously (Shang *et al.*, 2010), was used for the ChIP assay. To determine quantitatively WRKY40 binding to the *LHCB*promoters, real-time PCR analysis was performed according to a procedure described previously with the *Actin2* 3′-untranslated region sequence as the endogenous control (Mukhopadhyay *et al.*, 2008; Shang *et al.*, 2010). The primers used for real-time PCR analysis for different promoters are listed in Supplementary Table S2 at *JXB* online.

### Gel shift assay

**Fig. 4.**
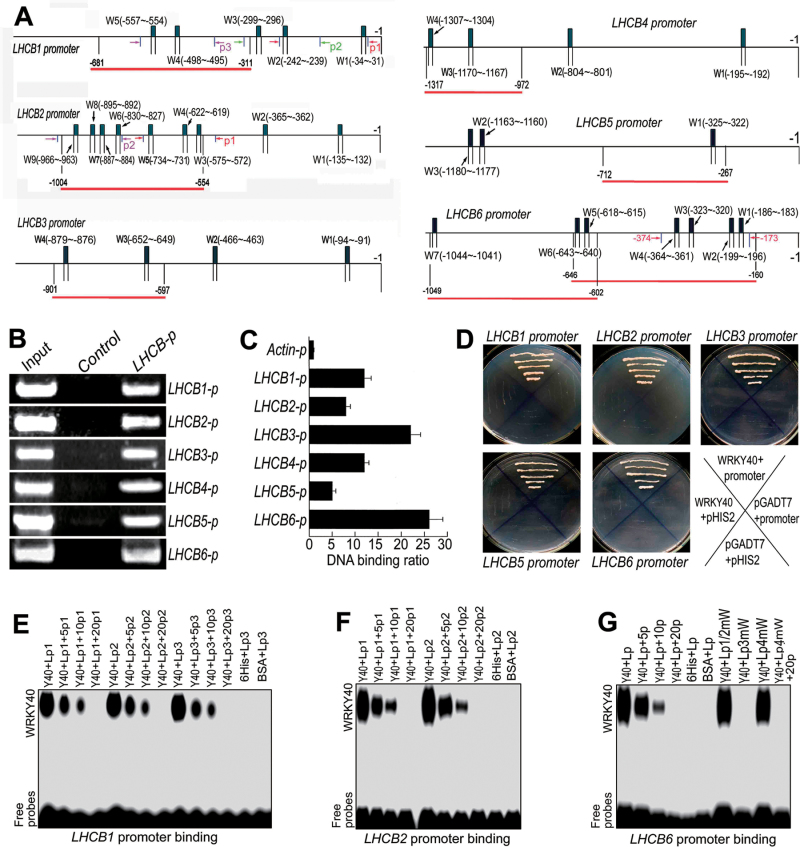
Transcription repressor WRKY40 binds the promoters of the members of the *LHCB* family. (A) The promoter structure of the *LHCB1–LHCB6* genes. W*n* (W1, W2, etc.) indicates W-boxes numbered from left to right and with their sequence sites relative to the translation start codon (ATG). Red linesindicate the sequences detected by ChIP assays described in (B). Arrows indicate the sequence fragments used inthe GSAs: the same fragment is indicated by two arrows of identical colour and p1, p2, etc. indicate numbering of the fragments. (B) WRKY40 interacts with the promoters of the *LHCB1–LHCB6* genes: PCR data from ChIP assays with the WRKY40-specific antibody (antibody againstWRKY40N). In the promoter fragment names, the suffix ‘*p*’ indicates promoter. The sequences for each promoter fragment are indicated in (A) and listed in detail in Supplementary Table S2. Lanes:Input, PCR product from the chromatin DNA;Control, PCR product from ChIP with pre-immune serum (as a negative control); *LHCB-p*, PCR product from ChIP with the antibody against WRKY40N. (C) WRKY40 interacts with the promoters of the *LHCB1–LHCB6* genes: real-time PCR data from the ChIP assay with the antibody against WRKY40N with the *Actin* promoter (*Actin-p*) as a negative control. The sequences for each promoter fragment are indicated in (A) and listed in detail in Supplementary Table S2. The symbols forpromoters present the same significances as described in (B). Each value is the mean ±SEM of three independent biological determinations. (D) WRKY40 interacts with the promoters of the *LHCB1–LHCB6* genes: yeast one-hybrid assay. The prey vector harbouring *WRKY40* (pGADT7-*WRKY40*,indicated by WRKY40) and the bait vector pHIS2 harbouring different *LHCB* promoters were used to transform yeast cells. Transformation with empty vectors pGADT7 and pHIS2 was used as negative controls. The experiments were repeated three times with the same results. (E–G) GSA showing that WRKY40 bindsthe promoters of the *LHCB1*(E), *LHCB2* (F), and *LHCB6* (G) genes. Y40, purified 6His–WRKY40 fusion protein;Lp, labelled promoter probe; p1, p2, etc. the non-labelled fragment described in (A); 5p, 10p, and 20p, 5-, 10-, and 20-fold unlabelled probe addition, respectively. Lp1/2mW, Lp3mW, and Lp4mW in (G) indicate the *LHCB6* promoter fragment with mutations in, respectively, the first and second-combined, third and fourth W-boxes (W1, W2, W3, and W4 indicated in A). Negative controls were a 6His tag peptide (6Hi) and bovine serumalbumin (BSA). The probe sequences are listed in detail in Supplementary Table S3. The experiments were repeated three times with the same results.

A gel shift assay (GSA) was performed using recombinant His–WRKY40 protein purified from *Escherichia coli* as described previously (Shang *et al.*, 2010). The promoter fragments used for the GSA were amplified by PCR using the following primer pairs: forward primer 5′-CATAACTTGTGGTCACAAAAC-3′ and reverse primer 5′-TTATGACTAACTTGTGAGTGAG-3′ for the first fragment of the *LHCB1* promoter (*pLHCB1*-1; –253 to –28, 226bp); forward primer 5′-AAGTTTTAGTTATTGGGTTGTA-3′ and reverse primer 5′-CATTCATTGGATTTTAAGAT-3′ for the second fragment of the *LHCB1* promoter (*pLHCB1*-2; –336 to –132, 205bp); forward primer 5′-GATAAAGAGTAAAACGTCAAAG-3′ and reverse primer5′-GTAACATTATAAAAAGCATTTACC-3′ for the third fragment of the 1 *LHCB1* promoter (*pLHCB1*-3; location in the promoter: –572 to –390; 183bp); forward primer 5′-TCTCTACCATTATGTGACTCTTG-3′ and reverse primer 5′-GCATGATTCGCTATGTCACAC-3′ for the first fragment of the *LHCB2* promoter (*pLHCB2*-1; –748 to –558, 191bp); forward primer 5′-CTATTACAACCGTTTAATTGAACC-3′ and reverse primer 5′-GCTTAGGTCATGAGCCATTAC-3′ for the second fragment of the *LHCB2* promoter (*pLHCB2*-2; –1010 to –821, 190bp), and forward primer 5′-ATTCATTGCTGTCATTTACATTTC-3′ and reverse primer 5′-GATAGATTTCTGACCAATTAGGAG-3′ for a fragment of the *LHCB6* promoter (*pLHCB6*; –374 to –173, 202bp). The suffix numbers of the designated fragment names correspond to the fragment numbers presented in Supplementary Table S3 at *JXB* online and in Fig. 4. The sequences amplified by these primer pairs are listed in Supplementary Table 3. The site-specific mutations of GTCA→GTTA or TGAC→TTAC in the core sequence of the W-box of the *LHCB6* promoter were introduced into the *LHCB6* promoter by two independent PCRs with the following primers (with the mutated W-box underlined) in addition to the above-mentioned primers for each promoter: forward primer 5′-ATTCATTGCTGTCATTTACATTTC-3′ and reverse primer 5′-GATAGATTTCTAACCAATTAGGAGTTAG-3′ for the mutated W-boxes W1 (GTCA→GTTA) and W2 (TGAC→TTAC); forward primer 5′-AATTTCCACGTGTTATTTTATTTTCC-3′ and reverse primer 5′-GATAGATTTCTGACCAATTAGGAG-3′ for the mutated W-box W3 (GTCA→GTTA), and forward primer 5′-ATTCATTGCTGTTATTTACATTT-3′ and reverse primer 5′-GATAGATTTCTGACCAATTAGGAG-3′ for the mutated W-box W4 (GTCA→GTTA). The locations of the W-box W1–W4 in the *LHCB6* promoter are indicated in Fig. 4A. Reconstitution was done using equimolar quantities of the two fragments from the initial PCRs for each promoter, which were used as templates for a third PCR. The mutations were verified by sequence analysis. Each of the promoter fragments was labelled on the base T with digoxigenin–dUTP (Roche, Mannheim, Germany) according to the manufacturer’s instructions. Binding reactions were performed as described previously (Shang *et al.*, 2010) using 50ng of His–WRKY40 fusion protein and 26ng for each of the digoxigenin-labelled promoter fragments. Competition experiments were performed using a 5- to 20-fold molar excess of unlabelled fragments.

### *Trans*-inhibition of LHCB promoter activity by WRKY40 in tobacco leaves

This assay was performed essentially as previously described (Shang *et al.*, 2010). WRKY40 was used for the effector construct. The cDNA of *WRKY40* was PCRamplified using forward primer 5′-CGCGGATCCATGGATCAGTACTCAT-3′ and reverse primer 5′-CCGCTCGAGCTATTTCTCGGTATGA-3′,and the PCR product was fused to the pBI121 vector downstream of the cauliflower mosaic virus 35S promoter at the *BamH*I/*Xho*I sites. Reporter constructs were composed of the *LHCB*promoter linked to the luciferase reporter gene (*LUC*). The *LHCB* promoters were isolated using the following primers: forward primer 5′-GGGGTACCCGCAGGGGAAAGGTTCACAG-3′ and reverse primer 5′-TCCCCCGGGTGCTTCGTGGAA AGTGATGC-3′ (976bp) for the *LHCB1* promoter; forward primer 5′-GGGGTACCGACGCCCACCTTTTGGATG-3′ and reverse primer 5′-TCCCCCGGGGGATTATTTGGATGGAT CATTTGG-3′ (1546bp) for *LUC LHCB2* promoter; forward primer 5′-GGGGTACCGAGAGCACTAAAGGCAAAGGACG-3′ and reverse primer 5′-TCCCCCGGGGCCAAGGAATGTTGTT GGGGTAA-3′ (1073bp) for *LUC LHCB3* promoter; forward primer 5′-GGGGTACCTGGTCTTGGATTTGGAGCTGG-3′ and reverse primer 5′-TCCCCCGGGCATTTCCGACACACCCAAAGAC-3′ (1384bp) for *LUC LHCB5* promoter; forward primer 5′-GGGGTACCTCCCGTGACTTTGCCTCCA-3′ and reverse primer 5′-TCCCCCGGGTCCGGTGAGGAACGAAGAAC-3′ (1109bp) for *LUC LHCB6* promoter. The *LUC* cDNA was PCRamplified using forward primer 5′-TCCCCCGGGATGGAAG ACGCCAAAAAC-3′and reverse primer 5′-CGGGATCCTTAC ACGGCGATCTTTCCGC-3′ from the pGL3-Basic vector harbouring the *LUC* cDNA. The DNA sequence of each *LHCB*promoter was fused to the *Kpn*I/*Sma*I sites of the pCAMBIA1300 vector, with the *LUC* cDNA fused to the *Sma*I/*BamH*I sites downstream of the *LHCB*promoters. The constructs were mobilized into *Agrobacterium tumefaciens* strainGV3101. Bacterial suspensions were infiltrated into young but fully expanded leaves of 7-week old *N. benthamiana* plants using a needleless syringe. The amount of constructwas the same among treatments and controls for each group of assay. After infiltration, plants were grown in the dark for 12h and then with 16h light per day for 60h at room temperature, and the LUC activity was observed with a CCD imaging apparatus (Andor iXon; Andor, UK). The experiments were repeated independently at least five times with similar results.

### Analysis of gene expression by promoter–β-glucuronidase (GUS) transformation

A promoter fragment of the *Arabidopsis* gene At1g15820 (*LHCB6*) was amplified by PCR using forward primer 5′-CCCAAGCTTCCGGACATGGGTTCAAATCA-3′ and reverse primer 5′-CGGGATCCAACCAAGCCCACTGAGGACA-3′. The DNA fragment was cloned into the pCAMBIA1391 vector and introduced into *Agrobacterium tumefaciens* strain GV3101 and transformed into *Arabidopsis* wild-type (Col-0) plants or *wrky40* mutant or *wrky40 wrky18* double mutant plants by floral infiltration. T3 generation homologous plants were used for the analysis of GUS activity. GUS staining was performed essentially according to Jefferson *et al.* (1987).

### Phenotypic analysis

Phenotypic analysis was done as described previously (Wu *et al.*, 2009,2012; Shang *et al.*, 2010). For germination assays, ~100 seeds were sterilized and planted in triplicate on MS medium (Sigma; full-strength MS). The medium contained 3% sucrose and 0.8% agar (pH 5.9) and was supplemented with or without different concentrations of ABA. The seeds were incubated at 4 °C for 3 d before being placed at 20 °C under light conditions, and germination (emergence of radicals) was scored at the indicated times. Seedling growth was assessed by directly planting the seeds in ABA-containing MS medium to investigate the response of seedling growth to ABA after germination.

### Accession numbers

Sequence data from this article can be found in the *Arabidopsis* Genome Initiative database under the following accession numbers: At5g13630 (*ABAR/CHLH*), At1g29920 (*LHCB1*), At2g05070 (*LHCB2*), At5g54270 (*LHCB3*), At2g40100 (*LHCB4*), At4g10340 (*LHCB5*), At1g15820 (*LHCB6*), At4g31800 (*WRKY18*), and At1g80840 (*WRKY40*). Germplasm identification numbers for mutant lines and SALK lines are: *aba2* (CS156: *aba2*-*1*), *abi5* (CS8105: *abi5-1*), *lhcb1.1* (*lhcb1*, SALK-134810), *lhcb2.2* (*lhcb2*, SALK-005614), *lhcb3* (SALK-036200), *lhcb4.4* (*lhcb4*, SALK-032779), *lhcb5* (SALK-139667), *lhcb6* (SALK-074622), *wrky40-1* (stock number: ET5883, Cold Spring Harbor Laboratory gene and enhancer trap lines), and *wrky18-1* (SALK_093916).

## Results

### Downregulation or disruption of *LHCB* genes reduces ABA responsiveness in seed germination and post-germination growth

**Fig. 1.**
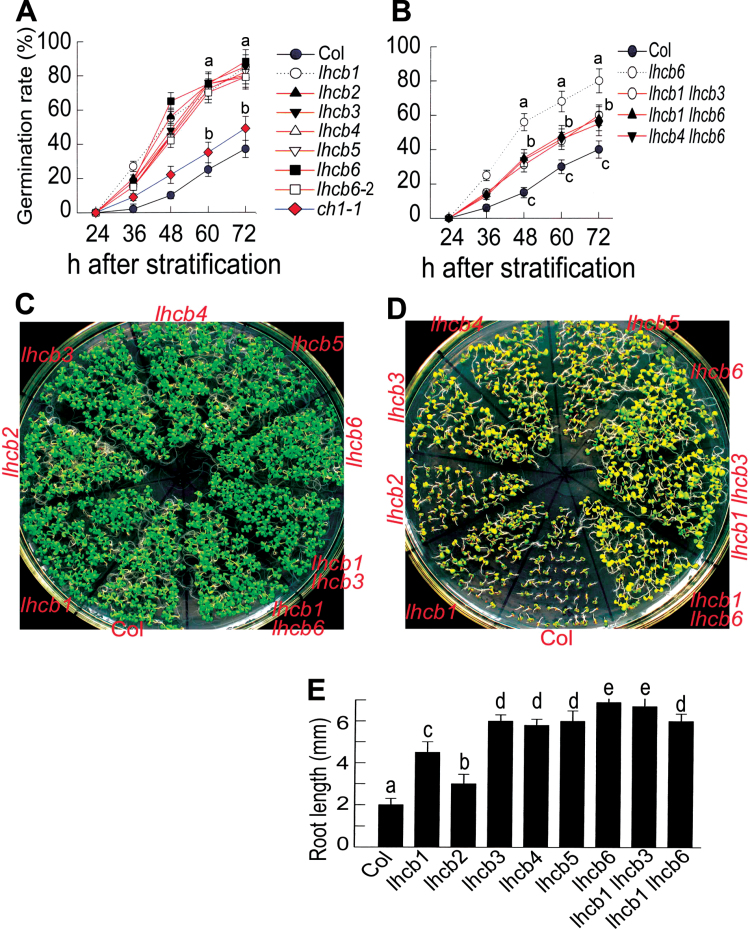
Downregulation of any member of the *LHCB* family reduces ABA sensitivities in seed germination andpost-germination growth. (A, B) Seed germination. Seed germination rate of the different *lhcb* single mutants(A) and *lhcb1 lhcb3*, *lhcb1 lhcb6*, and *lhcb4 lhcb6* double mutants (B) was assayed in 3 µMABA-containingmedium at the indicated time points after stratification. The wild-type Col-0 and the chlorophyll *b*-deficient *ch1-1*mutant were used as controls. *lhcb6-2*indicates the seeds of the *lhcb6* mutant harvested at a different time point.(C, D) Early seedling growth of the different *lhcb* single mutants and*lhcb1 lhcb3* and *lhcb1 lhcb6* double mutants in ABA-free (C) and 1 µMABA-containing (D) medium. The seeds were directly planted in theABA-free or ABA-containing medium, and observation was carried out at 12 d after stratification. (E) Quantitativedata of root length of the different genotypes in the 1 µMABA-containing medium as described in (D). Results in (A), (B), and (E) are means ±SEM of three independent biological determinations and the different letters indicate significant differences at *P*<0.05 (Duncan’s multiple-range test) when comparing values within thesame time point (A, B) or among the different genotypes (E).

We used the *lhcb1*, *lhcb2*, *lhcb4*, *lhcb5*, and *lhcb6* knockdown mutant alleles and the *lhcb3* knockout mutant allele to investigate whether LHCB members are involved in the regulation of seed germination and post-germination growth in response to ABA. These mutants were identified in our previous report (Xu *et al.*, 2012). We observed that all the *lhcb* single mutants displayed ABA-insensitive phenotypes in ABA-induced inhibition of seed germination and post-germination growth arrest, although the ABA-insensitive phenotypes in ABA-induced post-germination growth arrest were relatively weak (Fig. 1). These data revealed that the LHCB members are positive regulators of ABA signalling in these developmental processes. Unexpectedly,however, the double mutants*lhcb1 lhcb3*, *lhcb1 lhcb6*, and *lhcb4 lhcb6* showed weaker ABA-insensitive phenotypes than the *lhcb* single mutant (*lhcb6* for example) in ABA-induced inhibition of seed germination (Fig. 1B).

A chlorophyll *b*-deficient mutant,*ch1-1*,was used to assess the relationships between chlorophyll deficiency and ABA responsiveness. This *ch1* mutant showed a slight or no ABA insensitivity in seed germination and post-germination growth (Fig. 1A), indicating that the altered ABA-related phenotypes in the *lhcb* mutants were not caused by chlorophyll deficiency.

### *LHCB* expression is stimulated by physiologically high levels of ABA

To understand the underlying mechanism of the LHCB-mediated ABA signalling, we performed a detailed analysis to test the effects of ABA on *LHCB* gene expression. Previous studies focused generally on one member of the *LHCB* genes to assess the effects of ABA on *LHCB* expression (Bartholomew *et al.*, 1991; Chang and Walling, 1991; Weatherwax *et al.*, 1996; Staneloni *et al.*, 2008). We investigated all six members/representatives of the *Arabidopsis LHCB* genes (Jansson, 1999). The plants were treated with ABA using two different methods: for the first method, 3-d-old seedlings were grown for 2 weeks in medium containing 0, 0.5, 1, 2, 3, 5, or 10 µM ABA, and for the second, 5-week-old plants (2 weeks in MS medium plus 3 weeks in soil) were sprayed with ABA solution containing 0, 20, 50, 100, 150, 200, or 300 µM ABA, and sampled 5h after spraying for analysis. First, we assayed endogenous ABA concentrations in the treated plants to determine the enhanced range of endogenous ABA levels by exogenous ABA application. The endogenous ABA concentrations of the 3-d-old plants growing for 2 weeks in the medium containing 1, 3, 5, or 10 µM ABA increased, respectively, by 3-, 5-, 7-, and 12-fold relative to the ABA level of the plants growing in the ABA-free medium (Supplementary Fig. S1C at *JXB* online.). The endogenous ABA concentrations of the 5-week-old plants sprayed with ABA solution containing 50, 100, or 300 µM ABA increased, respectively, by about 45-, 60-, and 100-fold relative to the ABA level of the plants sprayed with the ABA-free solution (Supplementary Fig. S1B). We further assayed ABA levels of plants subjected to drought treatment under the environmental conditions of our experiment, and observed that a mild water stress could increase ABA levels by about 8- to 30-fold in comparison with the ABA concentrations in well-watered plants, and a severe drought could increase ABA levels by about 38- to 45-fold (Supplementary Fig. S1A). Thus, we could consider that the endogenous ABA levels of the 3-d-old seedlings growing for 2 weeks in medium containing 0.5–10 µM ABA and those of the 5-week-old plants sprayed with ABA solution containing 20 and 50 µM ABA didnot exceed the physiological limit of endogenous ABA concentrations, but that the endogenous ABA concentrations of the 5-week-old plants sprayed with ABA solution containing >100 µM ABA (100, 150, 200, or 300 µM) resulted in excessive ABA levels that went beyond the physiological limit of endogenous ABA concentrations.

**Fig. 2.**
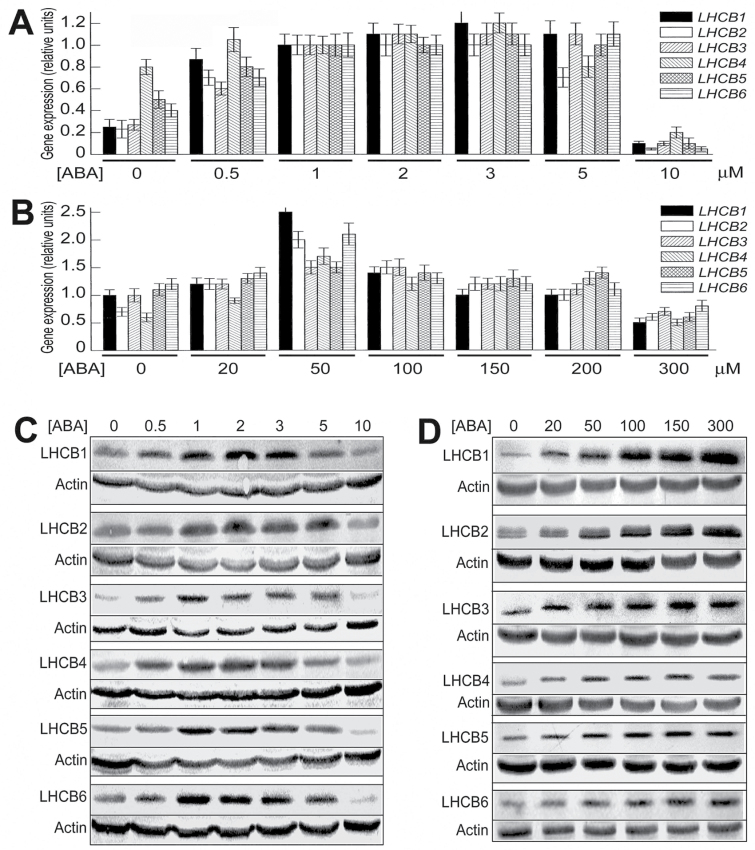
Low levels of ABA stimulate, but high levels of ABA inhibit, expression of *LHCB* genes. (A) In young seedlings, ABA treatments of 0.5–5 µM increased, but 10 µM decreased, mRNA levels of *LHCB1–LHCB6*. Three-day-old seedlings were transferred to ABA-containing MS medium and continued to grow 2 weeks before sampled for analysis. (B) In 5-week-old mature plants, ABA treatments of<200 µM increased, but >200 µM decreased, mRNA levels of *LHCB1–LHCB6*. Soil-grownplants were sprayed with ABA solution and sampled 5h later for analysis. (C) In young seedlings as described in(A), ABA treatments of 0.5–5 µM increased, but 10 µM decreased, the levels of LHCB1–LHCB6. (D) In mature plants as described in (B), ABA treatments of 20–300 µM increased the levels of LHCB1–LHCB6. In (A) and (B), each value is the mean ±SEM of three independent biological determinations. In (C) and (D), actin was used as a loading control and the experiment was replicatedthree times with similar results.

We observed that, with the first method whereby plants were grown for 2 weeks in ABA-containing medium from a young stage (3 dold), ABA treatments of 0.5–5 µM increased, but 10 µM decreased, the mRNA levels of the different *LHCB* members *LHCB1*–*LHCB6* (Fig. 2A), and it was noted that *LHCB4* expression was not significantly stimulated by 5 µM ABA treatment (Fig. 2A). The responses of the LHCB protein levels to ABA treatments were globally similar to those of the *LHCB* mRNA levels, with the highest stimulating effects of ABA at 1–3 µM (Fig. 2C). Also, we observed that the expression of all *LHCB* members except for *LHCB4* was upregulated by 5 µM ABA treatment at both mRNA and protein levels when 6-d-old seedlings were transferred to ABA-containing MS medium for a period of 24h (Supplementary Fig. S2 at *JXB* online.), which is essentially consistent with the observations of the 3-d-old plants grown for a longer time (2 weeks) in ABA-containing medium (Fig. 2A, C).

With the second method treating plants during the mature stage, ABA treatments for 5h at <200 µM increased, but at >200 µM decreased, the mRNA levels of the different members of the *LHCB* genes, with 50 µM ABA as the optimum concentration for stimulating the *LHCB* genes (Fig. 2B). At the protein level, ABA treatments of 20–300 µM enhanced the LHCB protein levels, and the stimulating effects were increased with increasing ABA concentrations (Fig. 2D), which is different from the effects on the *LHCB* mRNA levels (Fig. 2B), suggesting that LHCB expression is regulated differently at transcription and translation levels.

To test whether the exogenous ABA application affected expression of other genes encoding photosystem-related proteins, especially proteins involved in photosystem I (PSI) function, we measured, using the same methods of ABA treatments, mRNA levels of the genes encoding the LHCB proteins (LHCA1, LHCA2, LHCA3, and LHCA4) of PSI (Jansson, 1994, 1999), two subunits of the PSI core complex, the A/B (psaA and psaB, two highly homologous proteins) and D(psaD, including two highly homologous members psaD1 and psaD2) subunits of PSI (Büttner *et al.*, 1992; Scheller *et al.*, 2001; Knoetzel *et al.*, 2002), the γ subunit of chloroplast ATP synthase (including two highly homologous members, atpC1 and atpC2; Inohara *et al.*, 1991) and a subunit of the cytochrome *b6f* complex petC (Yuri *et al.*, 2001). We did not observed significant changes in the mRNA levels of these genes in response to exogenous application of ABA under our experimental conditions (Supplementary Fig. S3 at *JXB* online), which supports the observation that ABA-induced expression of *LHCB*s is specific and reliable.

We further investigated the effects of ABA treatments on the protein levels of several core components of PSI and PSII reaction centre complexes, including the PSI thylakoid proteins PsaA–PsaH, PSI antenna proteins LHCA1–LHCA4, and the PSII thylakoid proteins D1 (PsbA), D2 (PsbD), CP43, CP47, and PsbO. We observed that the levels of the assayed PSII reaction centre proteins were not significantly changed by ABA treatments, and neither were the levels of the most assayed PSI proteins (PsaA–PsaG, and LHCB1 and LHCB3) except for PsaH, LHCA2 and LHCA4 (Supplementary Fig. S4 at *JXB* online.). The PsaH level was repressed, but LHCA2 and LHCA4 levels were enhanced, by the ABA treatments (Supplementary Fig. S4A, C). These data further support the suggestion that the observed ABA-induced increase inLHCB protein levels is specific and reliable, and that ABA may also induce changes in the levels of other PSI/PSII proteins besides LHCBs.

Taken together, these data essentially showed that low levels of ABA, which, however, correspond to physiologically high levels of ABA, induce, rather than inhibit, *LHCB* expression. It is noteworthy, however, that young seedlings appeared to be more sensitive to ABA than mature plants, as evidenced by the above-mentioned observation that the expression of *LHCB*s was inhibited by 10 µMABA treatment resulting in a endogenously enhanced level of ABA (Fig. 2A, C), which did not exceed the physiological limit of endogenous ABA concentrations, while for mature plants (5 weeks old), the endogenous ABA at high concentrations over the physiological limit in the plants sprayed with 100, 150, and 200 µM ABA stimulated *LHCB* expression, although the endogenous ABA at a concentration that matched the physiological limit in the plants sprayed with 50 µM ABA had an optimum stimulating effect on *LHCB* expression at the mRNA level (Fig. 2B).

### ABA is necessary for full expression of *LHCB* genes

**Fig. 3.**
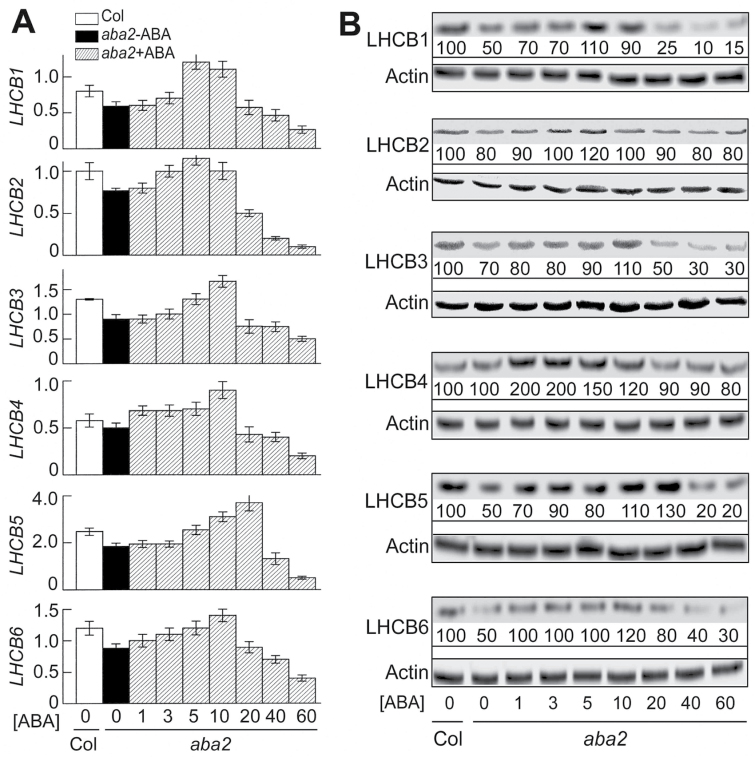
ABA is required for full expression of *LHCB* genes. (A) Expression of the *LHCB* genes, assayed by real-time PCR, was downregulated in the ABA-deficient mutant *aba2* plants, and ABA treatments could restore *LHCB* gene expression, but ABA treatments at higher concentrations (>40 µM) inhibited expression of these genes.Three-day-old mutant seedlings were transferred to ABA-free (0 µM; *aba2*–ABA) or ABA-containing medium (1–60 µM; *aba2*+ABA) and continued to grow for 2 weeks before being sampled for analysis. *LHCB1* to *LHCB6* indicate mRNA levels (normalized fold expression) of the corresponding *LHCB* genes. Col, Col-0 wildtype. Each value is the mean ±SEM of three independent biological determinations. (B) LHCB protein levels in the *aba2* mutant, and responses of the LHCB protein levels to ABA treatments (0, 1, 3, 5, 10, 20, 40, and 60 µM ABA) in the mutant.Three-day-old seedlings were treated as described in (A). Relative protein band intensities (%), normalized relative tothe intensity of Col-0 (with 0 µM ABA treatment; 100%), are indicated below the bands. Actin was usedas a loading control. The experiment was repeated three times with similar results.

We further showed that expression of the *LHCB* genes at both mRNA and protein levels was downregulated in the ABA-deficient mutant *aba2* plants except for LHCB4 for which the mRNA and protein levels were not reduced (Fig. 3A, B). ABA treatments could restores the mRNA and protein levels of the different LHCB members in the *aba2* mutant, but ABA treatments at higher concentrations (>20 or >40 µM for the *LHCB* mRNAs; and >20 µM for the LHCB proteins except for the LHCB5 protein: >40 µM) reduced both mRNA and protein levels of these LHCB members in the mutant (Fig. 3A, B). These findings demonstrated that ABA is required for full expression of the five *LHCB* members.

It is noteworthy, however, that the thresholds of ABA concentrations for inducing the responses of the *LHCB* expression increased significantly in the *aba2* mutant seedlings (Fig. 3A, B) in comparison with those in the wild-type seedlings (Fig. 2A, C).

### WRKY40 transcription factor binds the promoters of LHCB members and inhibits their expression

**Fig. 5.**
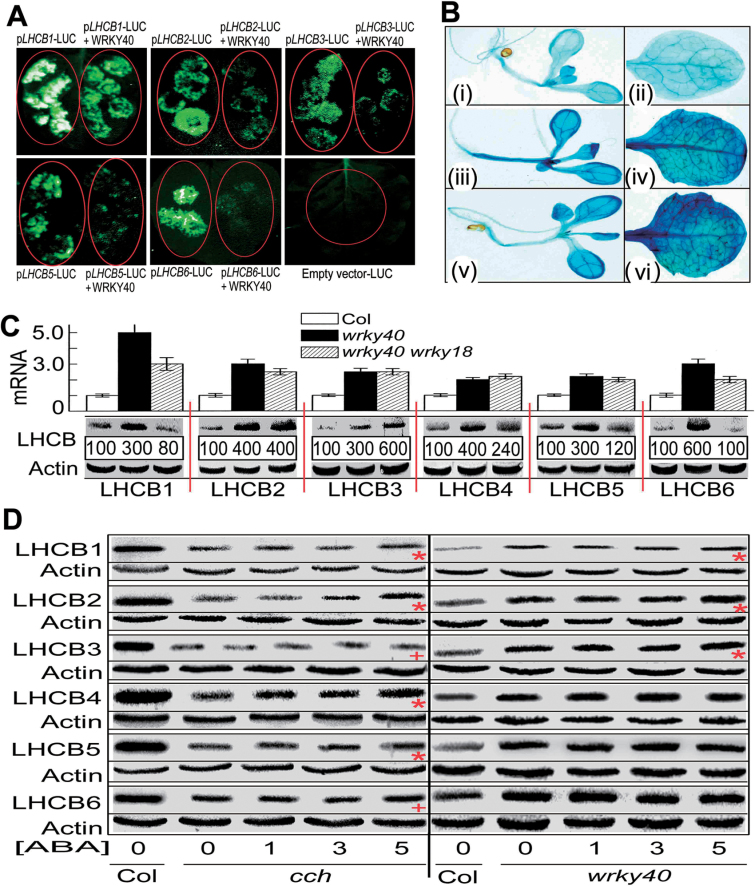
WRKY40 inhibits expression of *LHCB* genes. (A) WRKY40 inhibits the promoter activity of the *LHCB1*–*LHCB6* genes *in vivo*. Tobacco leaves were transformed with the constructs *pLHCB*–LUC alone and *pLHCB*–LUC plus *WRKY40*. The prefix ‘p’indicates promoter. Note that co-transformation ofWRKY40 and *pLHCB*–LUC reduced or even abolished *pLHCB*–*LUC* expression. The experiments were repeated three times with the same results. (B) *LHCB6* promoter-driven GUS expression in 3-d-old seedlings and mature leaves in the wild-type Col-0 (i, ii), *wrky40* single mutant (iii, iv) and *wrky40wrky18* double mutant (v, vi). Note that the *wrky40* and *wrky40 wrky18* mutations significantly enhanced the expression level of *LHCB6*. The experiments were repeated three times with the same results. (C)Expression of *LHCB1–LHCB6* is significantly upregulated in the *wrky40* single and *wrky40 wrky18*double mutants. mRNA was assayed by quantitative real-time PCR analysis (columns, indicated by mRNA), and protein was detected by immunoblotting (protein bands below the columns) with actin used as a loading control.Relative protein band intensities (%), normalized relative to the intensity of Col-0 (100%), are indicated below the bands. The immunoblotting assays were repeated three times with the independent biological experiments, which gave the similar results. Each value for real-time PCR is the mean ±SEM of three independent biological determinations. (D) Immunoblotting analysis showing that the stimulation of LHCB expression by ABA is partly dependent on the function of ABAR and WRKY40. Left panel: ABA treatment at 5 µM significantly (*P*<0.05, Duncan’s multiple range test) increases the protein levels of LHCB1, LHCB2, LHCB4, and LHCB5 (indicated by red asterisks) and slightly increases protein levels of LHCB3 and LHCB6 (indicated by red +) in the young seedlings of the *cch* mutant.Right panel: ABA treatment at 5 µM slightly increases the protein levels of LHCB1, LHCB2, and LHCB3 (indicated by red asterisks), but does not affect protein levels of LHCB4, LHCB5, and LHCB6 in the young seedlings of the *wrky40* mutant. Three-day-old seedlings were transferred to ABA-containing medium and continued to grow 2 weeks before being sampled for analysis. Actin was used as a loading control. The experiments were repeated three times with the same results.

To explore the mechanism by which ABA induces expression of the *LHCB* genes, we assessed whether a biotic stress- and ABA-responsive transcription factor, WRKY40 (Xu *et al.*, 2006; Shang *et al.*, 2010; Liu *et al.*, 2012; Yan *et al.*, 2013), regulated*LHCB* expression. With a combination of ChIP analysis, yeast one-hybrid assays, and GSA, we showed that WRKY40 binds the promoters of all these *LHCB* genes (Fig. 4). In the tobacco leaves co-transformed with both the *WRKY40*– and*LHCB*nativepromoter–LUC constructs, we observed that WRKY40 1 specifically inhibited expression of all these *LHCB* members *in vivo* (Fig. 5A). We introduced the *LHCB6*promoter-driven GUS into the *wrky40* single mutant and *wrky40 wrky18* double mutant, where WRKY18 co-operates with WRKY40 to regulate ABA signalling (Shang *et al.*, 2010; Liu *et al.*, 2012; Yan *et al.*, 2013), and found that the *wrky40* and *wrky40 wrky18* mutations significantly enhanced the expression level of *LHCB6* (Fig. 5B). We further showed that the mRNA levels of all six *LHCB* genes significantly increased in the *wrky40* single mutant and *wrky40 wrky18* double mutant, and the protein levels of all six LHCB members increased in the *wrky40* single mutant (Fig. 5C). In the *wrky40 wrky18* double mutant, however, the protein levels of LHCB2, LHCB3, LHCB4, and LHCB5 increased, while those of LHCB1 and LHCB6 decreased or did not change (Fig. 5C). Taken together, these findings are essentially consistent with a co-operative role of WRKY40 and its functional homologue WRKY18 in repression of *LHCB* genes.

### Mutations of ABAR and WRKY40 affect the responsiveness of LHCB expression to ABA

We observed that the levels of the LHCB proteins decreased significantly in the *cch* mutant, a mutant allele of the *ABAR* gene (Shen *et al.*, 2006; Wu *et al.*, 2009). We further showed that the protein levels of the LHCB members increased in response to the ABA treatments at low concentrations (1, 3, or 5 µM), but the strength of the ABA responsiveness declined significantly in the *cch* and *wrky40* mutants with no response of three LHCBs (LHCB3, LHCB4, and LHCB6) to ABA in the *wrky40* mutant (Fig. 5D). These data support the idea that ABA stimulates *LHCB* expression at least partly through the ABAR–WRKY40-coupled signalling pathway (Shang *et al.*, 2010).

### Downregulation of an LHCB member partly suppresses ABA hypersensitive phenotypes of the *wrky40* mutant

**Fig. 6.**
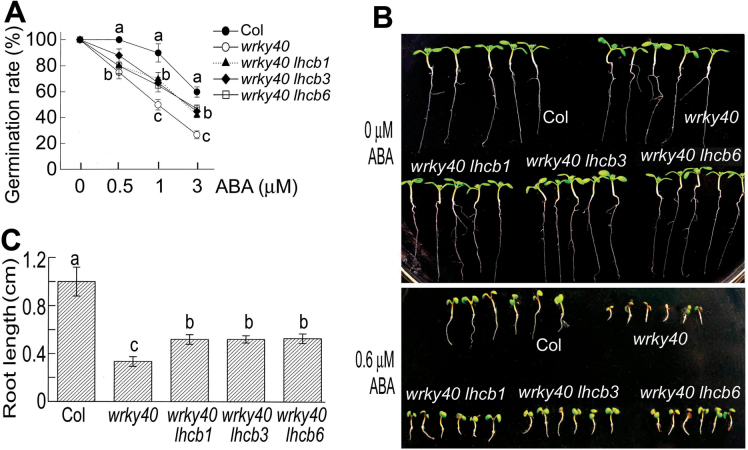
Downregulation of *LHCB6* expression reduces ABA hypersensitivity to partly restore wild-type ABA sensitivity of the *wrky40* mutant. (A) Downregulation of the *LHCB1*, *LHCB3*, and *LHCB6* expression reduces ABA hypersensitivity of the *wrky40* mutant in ABA-inhibited seed germination. The germination rates wererecorded 72h after stratification. (B) Downregulation of the *LHCB1*, *LHCB3*, and *LHCB6* expression reduces ABA hypersensitivity of the *wrky40* mutant in ABA-induced post-germination growth arrest. Seeds were directly planted in ABA-free (top panel) or 0.6 µMABA-containing (bottom panel) medium and the growth status was recorded 9 dafter stratification. (C) Quantitative data of root length in the 0.6 µM ABA-containing medium as described in (B). Each value in (A) and (C) is the mean ±SEM of three independent biological determinations and different letters indicate significant differences at *P*<0.05 (Duncan’s multiple range test) when comparing values within thesame ABA concentration (A) or among the different genotypes (C).

Previous studies showed that the *wrky40* mutant has ABA hypersensitive phenotypes in seed germination and post-germination growth (Shang *et al.*, 2010; Yan *et al.*, 2013). Introduction of the *lhcb1*, *lhcb3*, and *lhcb6* mutations into the *wrky40* mutant significantly reduced the ABA hypersensitive phenotypes of the *wrky40* mutant in seed germination and post-germination growth (Fig. 6). These data provided genetic evidence that the LHCBs function downstream of the WRKY40 transcription factor, consistent with the role of the LHCB members as direct targets of the WRKY40 transcription repressor (Figs 4 and 5).

## Discussion

### Positive role of LHCB members in the regulation of seed germination and post-germination growth in response to ABA

We reported previously that the members of the LHCB family positively regulate plant drought tolerance by functioning to positively control stomatal movement in response to ABA (Xu *et al.*, 2012). In the present report, we showed that the LHCB members positively regulate ABA signalling in seed germination and post-germination growth (Fig. 1). It is noteworthy that the *lhcb* double mutants showed ABA-insensitive phenotypes similar to or weaker than the *lhcb* single mutants (Fig. 1), suggesting that a compensatory feedback mechanism to maintain the LHCB homeostasis may function in the LHCB-related ABA signalling, as we proposed previously (Xu *et al.*, 2012). However, there may be other possibilities, for example that the significant decrease in the LHCB proteins in the double mutants may trigger a compensatory signalling events mediated by other components of ABA signalling than LHCBs, resulting in a partial rescue of ABA sensitivity in these double mutants. Further studies are needed to answer this question.

Each of the six *lhcb* single mutants showed similar ABA-insensitive phenotypes (Fig. 1), suggesting that each of the LHCB members is required for building the antenna complex and keeping the complex intact, which functions as a whole both in photosynthesis and ABA signalling. Deficiency of any of the LHCB members may damage this complex of the PSII antenna machinery, which affects ABA signalling. This is consistent with the point of view from the previous experiments where each member of the LHCB family plays a specific role in the regulation of the photosynthetic machinery and stomatal movement in response to ABA (Andersson *et al*., 2001, 2003; Ganeteg *et al.*, 2004; Kovacs *et al.*, 2006; Damkjaer *et al.*, 2009; Xu *et al.*, 2012).

It is well known that ABA induces stomatal closure in water-deficient conditions, which inhibits photosynthesis. Our previous report showed that LHCB proteins are positively involved in guard cell signalling in response to ABA in drought stress (Xu *et al.*, 2012). However, in the present experiment, we cannot answer the question of whether the ABA-induced accumulation of the LHCB proteins is favourable to photosynthesis. ABA-induced LHCB accumulation suggests possible changes in the levels of other photosystem/photosynthesis-related proteins.We observed that the mRNA levels of the assayed genes in the present experiment were not altered by exogenous ABA application (Supplementary Fig. S3). However,we showed that ABA treatments did not significantly change the levels of the assayed PSI and PSII proteins [PsaA–PsaG, LHCA1, LHCA3, D1 (PsbA), D2 (PsbD), CP43, CP47 and PsbO] except for PsaH, LHCA2, and LHCA4 (Supplementary Fig. S4). The mRNA levels of *LHCA2* and *LHCA4* did not changed by ABA treatments, suggesting that a translational or post-translational regulation may be involved in the ABA-induced increase in the LHCA2 and LHCA4 proteins. Given that the levels of most core components of PSI and PSII reaction centre complexes remained unchanged in response to ABA, we hypothesizethat the increase in the LHCB proteins in response to ABA may not function to regulate ABA signalling through fully functional antenna LHCB proteins involved in the PSII function. It will be interesting to assess how LHCB proteins act on ABA signallingin the future to understand the highly complicated ABA signalling pathway.

### ABA regulates expression of *LHCB* genes via the WRKY40 transcription repressor

Previous studies showed that exogenously applied ABA inhibits *LHCB* gene expression (Bartholomew *et al.*, 1991; Chang and Walling, 1991; Weatherwax *et al.*, 1996; Staneloni *et al.*, 2008). However, we noted that the ABA concentrations used in these studies should be much higher than physiological concentrations of ABA: ABA at 100 µM was applied to tomato leaves (Bartholomew *et al.*, 1991), at 300 µM to the 2-d-old *Arabidopsis* seedlings (Staneloni *et al.*, 2008), and at 10 µM to *L. gibba* grown on liquid medium (Weatherwax *et al.*, 1996). In the developing seeds of soybean, application of 50 µM ABA reduced *Cab3* (chlorophyll *a*/*b*-binding protein 3) expression, but 5 µM ABA treatment appeared to enhance the *Cab3* expression level (Chang and Walling, 1991). Interestingly, a recent report showed that the treatment of the 6-d-old *Arabidopsis* seedlings with low levels of ABA (from 0.125 to 1 µM) enhanced *LHCB1.2* mRNA levels (Voigt *et al.*, 2010). In the present experiments, we observed that expression of all six LHCB members in young seedlings was stimulated by exogenous application of ABA at low levels (Fig. 2,) resulting in enhanced internal ABA levels but within a natural range of physiologically high concentrations when ABA biosynthesis is induced by stresses. We found that the mature plants tolerated higher levels of exogenously applied ABA (Fig. 2), which may partly be due to a developmental stage-dependent response. Interestingly, in the ABA-deficient *aba2* mutant, we observed that ABA is required for full expression of all the *LHCB* genes except for *LHCB4* in both mRNA and protein levels (Fig. 3). The stimulation of LHCBs by physiological levels of ABA should be of particular functional significance, while ABA at higher-than-physiological levels may induce more complicated consequences to repress *LHCB* expression.

We further showed that the LHCB members are direct targets of an biotic stress- and ABA-responsive transcription repressor, WRKY40 (Xu *et al.*, 2006; Shang *et al.*, 2010; Liu *et al.*, 2012; Yan *et al.*, 2013), which is supported by several lines of evidence. First, the expression of *LHCB* genes was upregulated by the loss-of-function of *WRKY40* or double mutations in *WRKY40* and its closet functional homologue *WRKY18* (Fig. 5); secondly, all six LHCB members were clearly shown to be direct targets of the WRKY40 transcription factor that represses *LHCB* expression by using a combination of ChIP, yeast one-hybrid assays, GSAs, and co-transformation in a heterologous system (Figs 4 and 5); thirdly, the mutations in the *WRKY40* gene reduced responsiveness of the *LHCB* expression to exogenously applied ABA (Fig. 5); and lastly, downregulating expression of an LHCB member (*LHCB6*) partly suppressed the ABA-hypersensitive phenotype of the *wrky40* mutant (Fig. 6), which provides genetic evidence that LHCB proteins function downstream of WRKY40 in ABA signalling.

Additionally, we observed that the expression of *LHCB* genes was downregulated in an ABA-insensitive *abar* mutant allele, the *cch* mutant, which is opposite to what we observed in the *wyky40* mutant, and, in addition, the *cch* mutation reduced the responsiveness of *LHCB* expression to ABA (Fig. 5), revealing that *LHCB* expression requires a functional ABAR. These findings are consistent with the previously described working model that ABAR antagonizes the WRKY40 transcription repressor to relieve downstream ABA-responsive genes of inhibition (Shang *et al.*, 2010), and suggest that expression of the *LHCB* genes is controlled by the ABAR–WRKY40-coupled signalling pathway in response to ABA. We propose that, under non-stressful conditions, the homeostasis of the LHCB proteins is maintained by a complex signalling network where the WRKY40 transcription factor plays a negative role to balance the levels of the LHCB proteins. Under stressful conditions, the enhanced level of ABA represses the WRKY40 transcription repressor (Shang *et al.*, 2010) to relieve the *LHCB* genes of repression, which results in the ABA-related physiological responses.

Thus, the present experiments allowed us to identify the members of the LHCB family as novel targets of the biotic stress- and ABA-responsive WRKY40 transcription repressor (Xu *et al.*, 2006; Shang *et al.*, 2010). As LHCBs are important components of the photosynthetic machinery, expression of the *LHCB* genes are regulated essentially by light (Silverthorne and Tobin, 1984; Sun and Tobin, 1990; Peer *et al.*, 1996; Weatherwax *et al.*, 1996; Yang *et al.*, 1998; Humbeck and Krupinska, 2003; Nott *et al.*, 2006; Woodson and Chory, 2008; Staneloni *et al.*, 2008). We showed that ABA may be an inducer rather than a repressor used to fine-tune *LHCB* expression under stressful conditions in co-operation with light, which allows plants to adapt to environmental challenges.

## Supplementary data

Supplementary data are available at *JXB* online.

Supplementary Fig. S1. Endogenous ABA concentrations in plant tissues subjected to water stress or treated by exogenously applied ABA.

Supplementary Fig. S2. ABA at 5 µM stimulates expression of *LHCB* genes in 6-d-old seedlings grown in ABA-containing medium for 24h.

Supplementary Fig. S3. Exogenous ABA application does not change the expression of *LHCAs*, *psaA*, *psaD*, *petC*, or *atpC*.

Supplementary Fig. S4. Effects of exogenous ABA application on protein levels of the PSI and PSII proteins.

Supplementary Table S1. Primers used in this study.

Supplementary Table S2. Information for PCR and real-time PCR in ChIP assay.

Supplementary Table S3. Information for gel shift assays.

Supplementary Data

## Abbreviations:

ABA: abscisic acid
ABRC: *Arabidopsis* Biological Resource Center
ChIP: chromatin immunoprecipitation
GSA: gel shift assay
GUS: β-glucuronidase
LHCB: light-harvesting chlorophyll *a/b*-binding
LUC: luciferase
MS: Murashige–Skoog
PS: photosystem.
