# Supplementary material for: Light-harvesting chlorophyll a/b-binding proteins, positively involved in abscisic acid signalling, require a transcription repressor, WRKY40, to balance their function
Source: J Exp Bot. 2013 Sep 27;64(18):5443–56. doi: 10.1093/jxb/ert307 (PMC3871805; doi:10.1093/jxb/ert307)
Supplement: Supplementary Data [file supp_64_18_5443__index.html]

Light-harvesting chlorophyll a/b-binding proteins, positively involved in abscisic acid signalling, require a transcription repressor, WRKY40, to balance their function — Light-harvesting chlorophyll a/b-binding proteins, positively involved in abscisic acid signalling, require a transcription repressor, WRKY40, to balance their function — Supplementary Data 

# Light-harvesting chlorophyll *a*/*b*-binding proteins, positively involved in abscisic acid signalling, require a transcription repressor, WRKY40, to balance their function

## Supplementary Data

Data files

**Files in this Data Supplement:**

- Supplementary Data - Supplementary Data
