# Supplementary material for: Light-harvesting chlorophyll a/b-binding proteins, positively involved in abscisic acid signalling, require a transcription repressor, WRKY40, to balance their function
Source: J Exp Bot. 2013 Sep 27;64(18):5443–56. doi: 10.1093/jxb/ert307 (PMC3871805; doi:10.1093/jxb/ert307)
Supplement: Supplementary Data [file supp_ert307_jexbot105122_file001.pdf]

Supplementary Data

Liu R., Xu Y.H., Jiang S.C., *et al.*, Light-harvesting chlorophyll a/b-binding proteins, positively involved in abscisic acid signaling, require a transcription repressor WRKY40 to balance their function

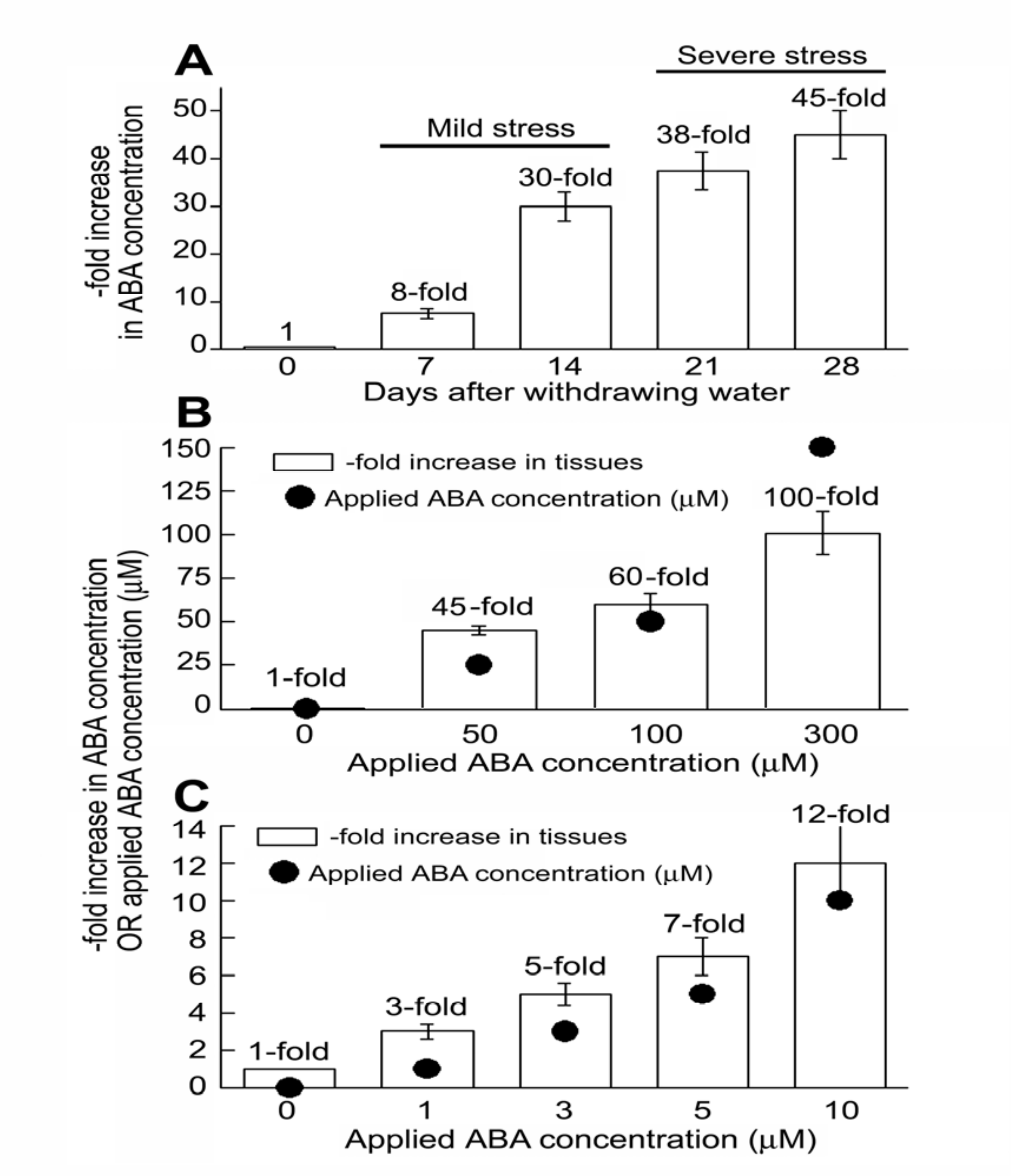

Supplementary Fig. S1. Endogenous ABA Concentrations in Plant Tissues Subjected to Water Stress (A) or Treated by Exogenously Applied ABA (B, C).

(A) Two-week-old wild-type Col plants were subjected to drought treatment (withholding water for 1 d, 7 d , 14 d,

21 d and 28 d, respectively), and the leaves sampled from these plants were used to assay ABA concentrations by ELISA method using a commercial kit (Plant Growth Regulator Immunoassay Detection Kit, PGR1, Sigma). The numbers mentioned above the columns indicate the relative values of ABA concentrations in stressed plants, normalized relative to the ABA concentration of well-watered or non-stressed plants (the relative ABA concentration was taken as 1, and the absolute value was about 0.7 to 0.8 μg/g dry weight). The plants began to wilt 14 d after withdrawing water, and wilted more and more severely thereafter, so the stress intensity between 7 and 14 d and that between 21 and 28 d after withdrawing water were considered, respectively, as "Mild stress" and "Severe stress". Each value is the mean ± SE of three independent biological determinations.

(B) Five-week-old Col plants (two weeks in MS medium plus three weeks in soil) were sprayed with ABA solution (0, 50, 100, or 300 μM), and sampled 5 h later for analysis of ABA concentrations in leaves by the same ELISA method as described above. The numbers mentioned above the columns indicate the relative values of ABA concentrations in ABA-treated leaves, normalized relative to the ABA concentration of ABA-free-treated leaves (the relative ABA concentration was taken as 1, and the absolute value was about 0.9 to 1.0 μg/g dry weight). Each value is the mean ± SE of three independent biological determinations.

(C) Three-day-old Col plants were grown two weeks in ABA-containing medium (0, 1, 3, 5, 10 μM), and the leaves sampled from these plants were used to assay ABA concentrations by the same ELISA method as described above. The numbers mentioned above the columns indicate the relative values of ABA concentrations in ABA-treated leaves, normalized relative to the ABA concentration of ABA-free-treated leaves (the relative ABA concentration was taken as 1, and the absolute value was about 0.4 to 0.5 μg/g dry weight). Each value is the mean ± SE of three independent biological determinations.

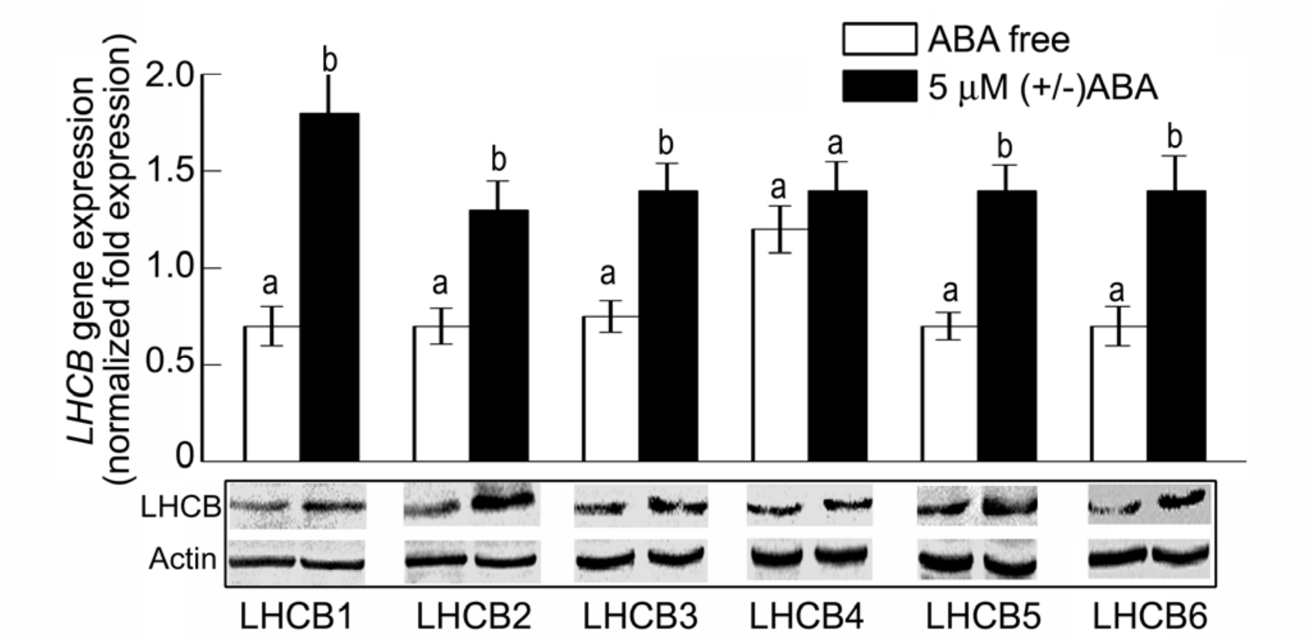

Supplementary Fig. S2. ABA at 5 μM Stimulates Expression of *LHCB* Genes in Six-day-old Seedlings Grown in ABA-containing Medium for 24 h.

Six-day-old seedlings were transferred to ABA-containing MS-medium for 24 h and sampled for analysis. ABA treatments at 5 μM increase both mRNA (top columns) and protein (bottom protein bands) levels of LHCBs (from LHCB1 to LHCB6) except for LHCB4. The mRNA and protein of LHCB4 is not significantly affected by ABA treatment. Top panel: Each value is the mean ± SE of three independent biological determinations and the different letters indicate significant differences at $P < 0.05$ (Duncan's multiple range test) when comparing values within the same gene. Bottom panel: Actin was used as a loading control; and the experiment was replicated three times with similar results.

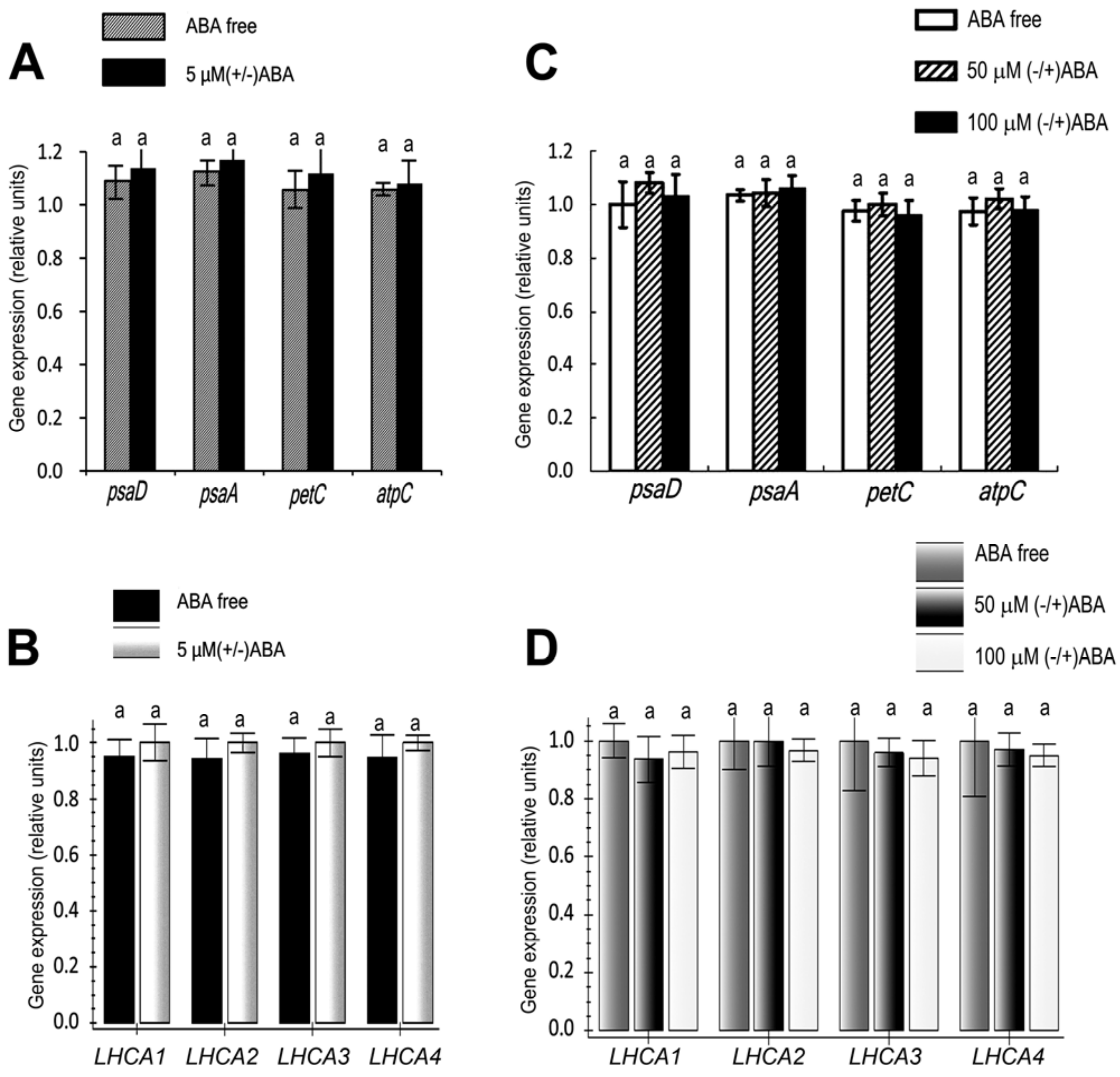

Supplementary Fig. S3. Exogenous ABA Application Does Not Change the Expression of *LHCAs*, *psaA*, *psaD*, *petC* or *atpC*.

(**A**) and (**B**) In young seedlings: ABA treatment at 5 μM did not change the mRNA levels of *psaA*, *psaD*, *petC*, *atpC* (A) or *LHCAs* (B). Three-day-old seedlings were transferred to ABA-containing MS-medium and continued to grow two weeks before sampled for real time PCR analysis.

(**C**) and (**D**) In five-week-old mature plants: ABA treatments at 50 or 100 μM did not change the mRNA levels of *psaA*, *psaD*, *petC*, *atpC* (C) or *LHCAs* (D). Soil-grown plants were sprayed with ABA solution and sampled 5 h later for real time PCR analysis.

The PCR primers for these genes are listed in Supplementary Table S1. Note that the primers for *psaA* cover tow homologous *psaA* and *psaB*, the primers for *psaD* cover tow homologous *psaD1* and *psaD2*, and the primers for *atpC* cover tow homologous *atpC1* and *atpC2*. Each value is the mean ± SE of three independent biological determinations and the same letters indicate non-significant difference at $P < 0.05$ (Duncan's multiple range test) when comparing values within the same gene.

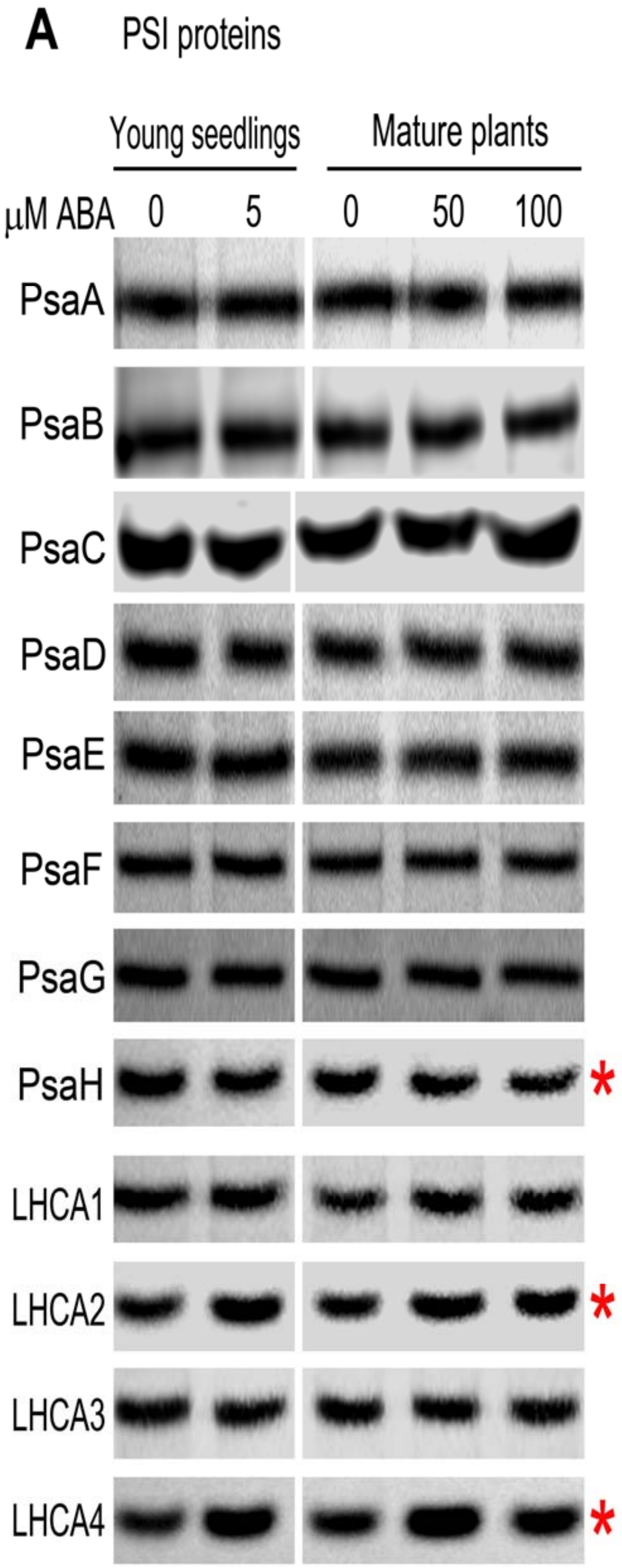

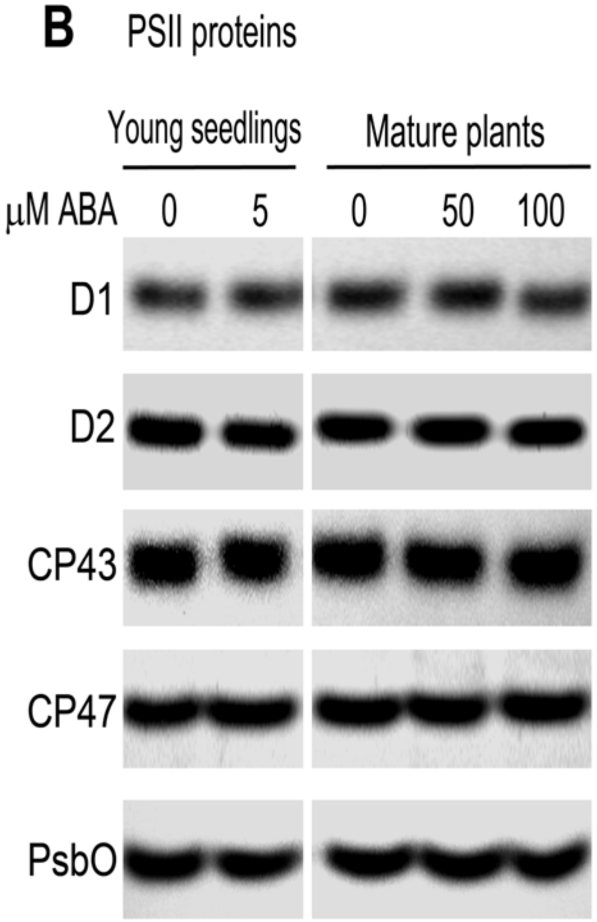

C

	ABA	PsaA	PsaB	PsaC	PsaD	PsaE	PsaF	PsaG	PsaH
Young	0	100±0a	100±0a	100±0a	100±0a	100±0a	100±0a	100±0a	100±0a
seedlings	5	100±0a	100±1a	97±5a	99±2a	100±2a	100±0a	100±0a	92±5b
Mature	0	100±0a	100±0a	100±0a	100±0a	100±0a	100±0a	100±0a	100±0a
plants	50	100±1a	98±3a	100±2a	100±1a	97±5a	96±6a	102±2a	90±4b
	100	98±4a	100±0a	100±0a	98±3a	100±0a	100±1a	100±2a	80±4c

	ABA	LHCA1	LHCA2	LHCA3	LHCA4	D1	D2	CP43	CP47	PsbO
Young	0	100±0a	100±0a	100±0a	100±0a	100±0a	100±0a	100±0a	100±0a	100±0a
seedlings	5	100±0a	122±11b	96±5a	187±20b	100±1a	100±2a	100±1a	99±2a	97±4a
Mature	0	100±0a	100±0a	100±0a	100±0a	100±0a	100±0a	100±0a	100±0a	100±0a
plants	50	100±2a	120±10b	100±1a	190±21c	100±0a	100±0a	100±0a	100±0a	100±0a
	100	104±5a	117±9b	100±0a	110±5b	100±0a	100±0a	100±0a	100±0a	100±0a

Supplementary Fig. S4. Effects of Exogenous ABA Application on Protein Levels of the PSI and PSII proteins. **(A)** ABA treatments (at 5 µM in young seedlings and at 50 µM or 100 µM in mature plants) did not change the protein levels of PsaA-PsaG and LHCA1 and LHCA3, but decreased PsaH level and enhanced LHCA2 and LHCA4 levels.

(B) ABA treatments (at 5 μM in young seedlings and at 50 μM or 100 μM in mature plants) did not change the levels of D1, D2, CP43, CP47 and PsbO proteins.

Treatments of young seedlings: Three-day-old seedlings were transferred to ABA-containing MS-medium and continued to grow two weeks before sampled for immunoblot analysis. Treatments of mature plants: Five-week-old soil-grown plants were sprayed with ABA solution and sampled 5 h later for immunoblot analysis. The antibodies were purchased from Agrisera. The immunoblot assays were conducted with loading of the equal total leaf proteins (15 μg). Five independent biological replicates were performed with substantially the same results, and a representative one is shown.

(C) Quantitative analysis of the data shown in the figures **A** and **B**. The numbers show the relative protein band intensities of the different proteins, where the protein band intensities of the ABA-treated samples were normalized relative to the intensity of the samples of ABA-free treatment (taken as 100%). Each value is the mean ± SE of five independent biological determinations and the different letters indicate significant differences at $P < 0.05$ (Duncan's multiple range test) when comparing values within young seedlings or mature plants. The red or pink numbers indicate the groups with significant changes in protein levels after ABA treatment.

Supplementary Table S1. Primers Used in this Study.

1. Gene-specific primers for real-time PCR analysis of gene expression

LHCB1:
forward primer: 5'-ATGGCCGCCTCAACAATGG-3'
reverse primer: 5'-CGGTAAGGTAGCTGGGTGAC-3'
LHCB2:
forward primer: 5'-ATGGCCACATCAGCTATCC-3'
reverse primer: 5'-CTCCAGTTAAGTAAGACGGTGTG-3'
LHCB3:
forward primer: 5'-AATGATCTTTGGTATGGACCTGAC-3'
reverse primer: 5'-CCACACGGACCCACTTTTG-3'
LHCB4:
forward primer: 5'-CAGCCGTACACTGAAGTCTTTGG-3'
reverse primer: 5'-TTCTATCCATATCAACGTCGTCAAC-3'
LHCB5:
forward primer: 5'-GGAGCAGCTGGTTTCATCATT-3'
reverse primer: 5'-ATCAAATGGACCTCCTGGG-3'
LHCB6:
forward primer: 5'-GCGATGGCAGCGGTTCTTG-3'
reverse primer: 5'-CCATGGCGTTGCCCACTCA -3'
LHCA1 :
Forward primer: TCTGCTCCTGGTGACTTTGGG
Reverse primer: TCCTGAGCCTTAACCCAGTTTCCAT
LHCA2 :
Forward primer: ATGGCATCATCTCTTTGTGCTTCTT
Reverse primer: CCGTCTAGCCACTCTGGAGGA
LHCA3:
Forward primer: TGTTGTTAAAGCTGCTGCA
Reverse primer: CTCTCCGTATGCTAGCCATCTTG
LHCA4:
Forward: primer: ATGGCTACTGTCACTACTCA
Reverse primer: AGATAATCAGGCGATGCCAAAC
psaA (covering tow homologous *psaA* and *psaB*):
Forward primer: GTCTATGGTTAACCGATATAGC
Reverse primer: GGCATGGAATACATATGGTGAGCTA
psaD (covering tow homologous *psaD1* and *psaD2*):
Forward primer: GCGATCATGAGAGAAGGTCCG
Reverse primer: CACCTTCTCTTCCTGGATTCGC
petC:
Forward primer: GCGTCCTCATCCCTTTCCCCTG
Reverse primer: AGAGAAAGAGCCCCAAGAAGAAG
atpC (covering tow homologous *atpC1* and *atpC2*):
Forward primer: TTGTGTCTTTGGTGAAATCTGA
Reverse primer: GCATCAAGAATCTGAACAGGGTC

2. Specific primers for PCR and real-time PCR analysis of promoters in ChIP assay

The prefix *p* indicates promoter.
pLHCB1:
forward primer: 5'- GTCTAAACCCATGATGATGAACA-3'
reverse primer: 5'-TGCTACAACCCAATAACTAAAACTT-3'
pLHCB2:
forward primer: 5'-CAACCGTTTAATTGAACCATTGC-3'
reverse primer: 5'-AGTGTGACATAGCGAATCATGCAAG-3'
pLHCB3:
forward primer: 5'-GGCTACTCAGTAGCAAAGACGA-3'
reverse primer: 5'-TTCTAGCGACCTCTCAAGCGG-3'
pLHCB4:
forward primer: 5'-CTTTCGGCGTTGACCCATC-3'
reverse primer: 5'-ACATTATATTAGTTAGGGCGATTT-3'
pLHCB5:
forward primer: 5'-CGATTCTACCGTAGTACTGTGTGTG-3'
reverse primer: 5'-AGATTCTTGTAACACATTTTGCTGT-3'
pLHCB6-1:
forward primer: 5'-TCCCGTGACTTTGCCTCCA-3'
reverse primer: 5'-ACCAATAGAGTCCATCCCAACAAT-3'
pLHCB6-2:
forward primer: 5'-GAGGTCACAAATCCATGGCAAC-3'
reverse primer: 5'-GAAATCTATCCACGACAGCCTC-3'

3. Primers for Cloning *LHCB*-Promoters for Yeast One-Hybrid Assay

LHCB1 promoter (946 bp):
forward primer: 5'-TCCCCCGGGGCTTCGTGGAAAGTGATGCAA-3'
reverse primer: 5'-CGACGCGTCGAAAGCAGGGGAGGAGAGAG-3'
LHCB2 promoter (1537 bp):
forward primer: 5'-TCCCCCGGGGGATTATTTGGATGGATCATTTGG-3'
reverse primer: 5'-CGACGCGTCTTTTGGATGAGGTCGCTGGAG-3'
LHCB3 promoter (672 bp):
forward primer: 5'-TCCCCCGGGCTAGCGACCTCTCAAGCGGAAC-3'
reverse primer: 5'-CGACGCGTCCAAGGAATGTTGTTGGGGTAAG-3'
LHCB5 promoter (1279 bp):
forward primer: 5'-TCCCCCGGGCCATCTCTGTGAGTATTTGTCTGC-3'
reverse primer: 5'-CGACGCGTGTACCAAGCATTTCCGACACAC-3'
LHCB6 promoter (1137 bp):
forward primer: 5'-TCCCCCGGGACCAGTCAACATTAACGCCACC-3'
reverse primer: 5'-CGACGCGTTCCGGTGAGGAACGAAGAAC-3'

Supplementary Table S2. Information for Detecting the WRKY40-Binding Sequences of the *LHCB* Promoters by PCR and Real-time PCR in the Chromatin Immuno-Precipitation Assay (ChIP) with the Antibody against the Truncated WRKY40^{N}.

The prefix *p* to gene name indicates fragmented promoter of the gene; Location, the fragment location site relative to the start codon ATG; Sequence, the sequence amplified by PCR using the presented primer pair (with the W-box indicated in **RED**). The primer pairs are marked in **PINK**.

pLHCB1: forward primer 5'-**GTCTAAACCCATGATGATGAACA**-3' and reverse primer 5'-**TGCTACAACCCAATAACTAAAACTT**-3'.

Location: -681 – -311.

Sequence (370 bp):

GTCTAAACCCATGATGATGAACATGCCATATGAATTAATATAGTTCTTTCATGTTCTTAGAAATGGTCGA
TAAGTTAAAACAGTGTTTCATTAATCGTTGTGTTTCTTCGATAAAGAGTAAAACGTCAAAGTTTTAAGGT
GATATCACATAATTCATGAGATTATTTTGGTTATTGGATAAAG**TGAC**GATGAAATCGATTCATAATTGAAAT
TAAACTGGTAGTGATGATGAACTAATTACCATTGAAAATATTGATATTTATGTGTGGGTAAATGCTTTTTAT
AATGTTACTTTATATTAATGTTTCGATCATCAGAATCTATATTTTCAAAATGTTATACTTT**AAGTTTTAGTTA
TTGGGTTGTAGCA**

pLHCB2: forward primer 5'-**CAACCGTTTAATTGAACCATTGC**-3' and reverse primer 5'-**AGTGTGACATAGCGAATCATGCAAG**-3'.

Location: -1004 – -554.

Sequence (450 bp):

CAACCGTTTAATTGAACCATTGCAATATTTAGTTTATT**TGAC**GAACCATTAAAATTATTGGTTTATTTTAT
GAACTCTTGTAATATTTTATATATAAATAGTAGCGATC**TGAC**TAAA**TGAC**TTATACGTTTGGGTTTCTCAAT
TTTGTAACTGTGTTTGCATTGTAATGGCTCA**TGAC**CTAAGCCTAATTTGGGAGGCCCATATATATAAAAAC
TCTGTAAGAAACAAGGTTCGAATTTGTAGCAAATGTAATCTTTCTCTACCATTATG**TGAC**TCTTGTAAGA
GCCATAGCCAAGGAAAATTACTTAAGTTAGGTAATGTTCTGTGAAATATATTTTTCCTCCTCTGTAAATAA
AGTGTGTTTTTCTATTTACTTGCAGTT**TGAC**TTCTTGATCTGGTAAGTGTTGGCTTTTAATATATGATTT**AG
TGTGACATAGCGAATCATGCAAG**

pLHCB3: forward primer 5'-**GGCTACTCAGTAGCAAAGACGA**-3' and reverse primer 5'-**TTCTAGCGACCTCTCAAGCGG** -3'.

Location: -901 – -597.

Sequence (304 bp):

GGCTACTCAGTAGCAAAGACGATGACGAGGACGAAGATGATGAAGACAGAGAAAAGAAGCTTAACA
AATCTCAGCCTCGGGTTTGTTAATCACATCTATTTTCCCTTCTTTCGCTGCTTATTAGCAGGTTTTAGTAAG
TTGTTGTTAACCATTTGAGATCAAAGCTCACTTAATAGTACAATTTGAATATGCAGGTTACAAGGAACTAT
GGAGCTCTAAGAAGATAAATACATATCAAACCCCAATCTC**TGAC**ATCACAACGAAGCTTCATTTTTCTGT
TATT**TTCTAGCGACCTCTCAAGCGG**

pLHCB4: forward primer 5'-**CTTTCGGCGTTGACCCATC**-3' and reverse primer 5'-**ACATTATATTAGTTAGGGCGATTT**-3'.

Location: -1317 – -972.

Sequence (345 bp):

CTTTCGGCGTTGACCCATCAACTCCTTCGCTTTTAAGTTTCAGGTAAACCAACCTAATCTCATCCTCTT
CCAATGTTCTTGCTTACGTATTTGTTATTCTTCTTCTTTTTTTGAATAGAAATGATTTCTATAATATTATT

AATTGGGAAG**TGAC**ATTATGGACGTTACAAAGTTGAGAACCCTTTTGCTGATAATGTTTGTTTATTTGT
CTTTTTTTTTTCTTTAACAGAAGGATCCAAAGCAAAAGGAATGGAGATTAATTATATAACCTATGCTTT
TTTAAATGAGACATTTCAGATGAACTGGCAGTTGTTAGCTTTA**ACATTATATTAGTTAGGGCGATTT**

pLHCB5: forward primer 5'-**CGATTCTACCGTAGTACTGTGTGTG**-3' and reverse primer
5'-**AGATTCTTGTAACACATTTTGCTGT**-3'.
Location: -712 – -267.
Sequence (445 bp):
CGATTCTACCGTAGTACTGTGTGTGAACCGGCACTGTGAACCAAGATGATTAAATTTTCGTATTCTCTA
TGTACATGATCCTGCGGCTCAATCGCTTCAGTTTCGATCCACATGATGTATATGTTATAGAATTGTGGGAA
ACTCCTTGTAGAAAGAGTATGTTCACGTCTAGGACTAGTCGGATGATTCGTTTCTCTTTTTGGTGTAATGA
GTATGTTCATAACTGTTGATACAATGTGAAAATCTAACCGTTGAGCTTGGGAGTTTTACGTCTATATGAAA
ATTCCGGTTGTCGTCTACATTACGGTAGTAAACAGGACCACAGTGATTCCAAATGTCCCAAGGAATTTAC
TGAAAACCCCAACTAGGACTGTGAAAGGCTTGTGGA**TGAC**ATTTAACATTGAGATTTTCATGTGTTTG**A**
GATTCTTGTAACACATTTTGCTGT

pLHCB6-1: forward primer 5'-**TCCCGTGACTTTGCCTCCA**-3' and reverse primer
5'-**ACCAATAGAGTCCATCCCAACAAT**-3'.
Location: -1049 – -578.
Sequence (471 bp):
TCCCGTGACTTTGCCTCCACCGGTTCAAGACCTTCCTTCGATTTTACCTCCCCCGGCTGATGAGTTGCC
GCCACCGGTTCAAGAATTCCCTCCGATTTTGCCTCCACCGGTTCAAGATTTCCCCCCAATTCTCGCTCCC
CCGGCTGATGAGTTCCCGCCAAATTTGCCTCCACCGGTTCTAGAATTCCCTCCGATTATGCCTCCACCGG
TTCAAGATTTCCCGCCAATTCTCACTCCACCGGCTGAAGAGTTCCCGCCGATTTTGCCTCCACCGGTTCA
AGAGATCCCGCCGGTTTTCACATTACCACCGACCGTACAAGATCCACCGACAATTCCAGTATTCTCCACA
CCACCAGTCCTCGGAGATTTCCCACCCCAAACTCCCGACTTTACCACGCCGCCAGAG**GTCA**CAAATCCA
TGGCAACCGCCGG**TGACGTCA**TTCGCACC**ACCAATAGAGTCCATCCCAACAAT**

pLHCB6-2: forward primer 5'-**GAGGTCACAAATCCATGGCAAC**-3' and reverse primer
5'-**GAAATCTATCCACGACAGCCTC**-3'
Location: -646 – -160.
Sequence (486 bp):
GAGGTCACAAATCCATGGCAACCGCCGG**TGACGTCA**TTCGCACCACCAATAGAGTCCATCCCAACAA
TACCGGATAATCCGTTTCCGGTTACACCAAACCCGGACATGGGTTCAAATCAACCGTTTGTTGAGCTTCC
TCCGCCTACTTGGGATTCCCCGCCATTTAATCGTTAATTCTCTTTATATATTTTTAAATTTTCGTTTTTTTTT
CAGTTAATCATGTAATGTGTAATCTAAACGCAATAAGCCACATAATGCAGCCATTTTGTAAATTCATTGCT
GTCATTTACATTTCAATAAGAAAATAAATTAATTTCCACGT**GTCA**TTTTATTTTCCTCTCACAATTTAGATT
TAGATAAGTAAAGTATTAGAAAAATGTAAATACTGAGTTGGCAGAATAAGACAGTGGTGAGTGTGGGTG
ATGTGGATATGGTGAAGTAGTTTC**TGAC**TCCTAATTG**GTCAGAAATCTATCCACGACAGCCTC**

Supplementary Table S3. Information for Analysis of the WRKY40-*LHCB* Promoter Binding by Gel Shift Assay. The prefix *p* to gene name indicates fragmented promoter of the gene; Location, the fragment location site relative to the start codon ATG; Sequence, the sequence amplified by PCR using the presented primer pair (with the W-box indicated in **RED**). The primer pairs are indicated in BLUE.

pLHCB1-1: forward primer 5'-CATAACTTGTG**GTCA**CAAAAC-3' and reverse primer
5'-TTATGACTAACTTGTGAGTGAG -3'.
Location: -253 – -28.
Sequence (226 bp):
CATAACTTGTG**GTCA**CAAAACGCTTGGCTGCAATGAAAAAATCAAAACAAATGCTGGTGGACTAGAGAT
TGCCACGTAAGACTACTAAACGATAAAACAAAAATCTTAAAATCCAATGAATGAACAGATAAAGATTACT
TCAGATATAACAAACGTTACAATATCCCTATATAATCCAACACTATCGAACCAGTTTTAATCACTCTCACTC
ACAAGTTA**GTCA**TAA

pLHCB1-2: forward primer 5'-AAGTTTTAGTTATTGGGTTGTA-3' and reverse primer
5'-CATTCATTGGATTTTAAGAT-3'.
Location: -336 – -132.
Sequence (205 bp):
AAGTTTTAGTTATTGGGTTGTAGCAAAAATCATTCTT**GTCA**CGAGGGTGTAAGTAAGTGTAACCGTTGAA
GTATTCAGTGGCTCATAACTTGTG**GTCA**CAAAACGCTTGGCTGCAATGAAAAAATCAAAACAAATGCTG
GTGGACTAGAGATTGCCACGTAAGACTACTAAACGATAAAACAAAAATCTTAAAATCCAATGAATG

pLHCB1-3: forward primer 5'-GATAAAGAGTAAAACGTCAAAG-3' and reverse primer
5'-GTAACATTATAAAAAGCATTTACC-3'.
Location: -572 – -390.
Sequence (183 bp):
GATAAAGAGTAAAACGTCAAAGTTTTAAGGTGATATCACATAATTCATGAGATTATTTTGGTTATTGGATA
AAG**TGAC**GATGAAATCGATTCATAATTGAAATTAAACTGGTAGTGATGATGAACTAATTACCATTGAAAA
TATTGATATTTATGTGTGGGTAAATGCTTTTTATAATGTTAC

pLHCB2-1: forward primer 5'-TCTCTACCATTATG**TGAC**TCTTG-3' and reverse primer
5'-GCATGATTCGCTAT**GTCA**CAC-3'.
Location: -748 – -558.
Sequence (191 bp):
TCTCTACCATTATG**TGAC**TCTTGTAAGAGCCATAGCCAAGGAAAATTACTTAAGTTAGGTAATGTTCTG
TGAAATATATTTTTCCTCCTCTGTAAATAAAGTGTGTTTTTCTATTTACTTGCAGTT**TGAC**TTCTTGATCT
GGTAAGTGTTGGCTTTTAATATATGATTTAGTG**TGAC**ATAGCGAATCATGC

pLHCB2-2: forward primer 5'-CTATTACAACCGTTTAATTGAACC-3' and reverse primer
5'-GCTTAGGTCATGAGCCATTAC-3'.
Location: -1010 – -821.
Sequence (190 bp):
CTATTACAACCGTTTAATTGAACCATTGCAATATTTAGTTTATT**TGAC**GAACCATTAAAATTATTGGTTT
ATTTTATGAACTCTTGTAATATTTTATATATAAATAGTAGCGATC**TGAC**TAAA**TGAC**TTATACGTTTGG
GTTTCTCAATTTTGTAACTGTGTTTGCATTGTAATGGCTCA**TGAC**CTAAGC

pLHCB6: forward primer 5’-ATTCATTGCTGTCATTTACATTTC-3’ and reverse primer 5’-GATAGATTTCTGACCAATTAGGAG-3’.
Location: -374 – -173.
Sequence (202 bp):
ATTCATTGCT**GTCA**TTTACATTTCAATAAGAAAATAAATTAATTTCCACGT**GTCA**TTTTATTTTCCTCTC
ACAATTTAGATTTAGATAAGTAAAGTATTAGAAAAATGTAAATACTGAGTTGGCAGAATAAGACAGTG
GTGAGTGTGGGTGATGTGGATATGGTGAAGTAGTTTC**TGAC**TCCTAATTG**GTCA**GAAATCTATC
